# Metabolic Engineering of *Corynebacterium glutamicum* for High‐Level Production of 1,5‐Pentanediol, a C5 Diol Platform Chemical

**DOI:** 10.1002/advs.202412670

**Published:** 2024-12-27

**Authors:** Yu Jung Sohn, Se‐Yeun Hwang, Haeyoung Lee, Subeen Jeon, Ji Young Park, Jaehyung Kim, Donghyuk Kim, Ki Jun Jeong, Sang Yup Lee, Jeong Chan Joo, Jin‐Byung Park, Si Jae Park

**Affiliations:** ^1^ Department of Chemical Engineering and Materials Science Graduate Program in System Health Science and Engineering Ewha Womans University Seoul 03760 Republic of Korea; ^2^ Department of Food Science and Biotechnology Ewha Womans University Seoul 03760 Republic of Korea; ^3^ School of Energy and Chemical Engineering Ulsan National Institute of Science and Technology (UNIST) Ulsan 44919 Republic of Korea; ^4^ Department of Chemical and Biomolecular Engineering Korea Advanced Institute of Science and Technology (KAIST) Daejeon 34141 Republic of Korea; ^5^ Metabolic and Biomolecular Engineering National Research Laboratory Systems Metabolic Engineering and Systems Healthcare Cross‐Generation Collaborative Laboratory Department of Chemical and Biomolecular Engineering (BK21 four) Korea Advanced Institute of Science and Technology (KAIST) Daejeon 34141 Republic of Korea; ^6^ BioProcess Engineering Research Center Center for Synthetic Biology KAIST Institute for the BioCentury KAIST Institute for Artificial Intelligence KAIST Daejeon 34141 Republic of Korea; ^7^ Department of Chemical Engineering Kyung Hee University Yongin 17104 Republic of Korea

**Keywords:** 1,5‐Pentanediol, carboxylic acid reductase, *Corynebacterium glutamicum*, enzyme engineering, NADH/NADPH optimization

## Abstract

The biobased production of chemicals is essential for advancing a sustainable chemical industry. 1,5‐Pentanediol (1,5‐PDO), a five‐carbon diol with considerable industrial relevance, has shown limited microbial production efficiency until now. This study presents the development and optimization of a microbial system to produce 1,5‐PDO from glucose in *Corynebacterium glutamicum* via the l‐lysine‐derived pathway. Engineering began with creating a strain capable of producing 5‐hydroxyvaleric acid (5‐HV), a key precursor to 1,5‐PDO, by incorporating enzymes from *Pseudomonas putida* (DavB, DavA, and DavT) and *Escherichia coli (*YahK). Two conversion pathways for further converting 5‐HV to 1,5‐PDO are evaluated, with the CoA‐independent pathway—utilizing *Mycobacterium marinum* carboxylic acid reductase (CAR) and *E. coli* YqhD—proving greater efficiency. Further optimization continues with chromosomal integration of the 5‐HV module, increasing 1,5‐PDO production to 5.48 g L^−1^. An additional screening of 13 CARs identifies *Mycobacterium avium* K‐10 (MAP1040) as the most effective, and its engineered M296E mutant further increases production to 23.5 g L^−1^. A deep‐learning analysis reveals that *Gluconobacter oxydans* GOX1801 resolves the limitations of NADPH, allowing the final strain to produce 43.4 g L^−1^ 1,5‐PDO without 5‐HV accumulation in fed‐batch fermentation. This study demonstrates systematic approaches to optimizing microbial biosynthesis, positioning *C. glutamicum* as a promising platform for sustainable 1,5‐PDO production.

## Graphical abstract

This study develops an efficient biosynthesis system for 1,5‐pentanediol (1,5‐PDO) in *Corynebacterium glutamicum*, an important compound for industrial applications. By engineering the l‐lysine degradation pathway, 5‐hydroxyvaleric acid (5‐HV) is synthesized from glucose as a key intermediate. Further optimization of the 5‐HV conversion module using a mutant enzyme (MAP1040_M296E) and the GOX1801 gene enhances 1,5‐PDO production, achieving 43.4 g L^−1^ in fed‐batch fermentation.

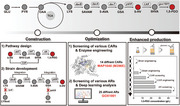

## 1 Introduction

The term “global boiling” is now used to describe current climate change, as recent climate data highlight a significant departure from the conventional understanding of global warming.^[^ ^1^ ^]^ This shift underscores the imperative of sustainable development, emphasizing the need to meet present needs without compromising the ability of future generations to meet their needs.^[^ ^2^, ^3^ ^]^ In line with this ethos, many metabolic engineers have directed their efforts toward developing efficient microbial cell factories capable of producing various value‐added products from sustainable resources. Among these, C5 platform chemicals, including 5‐aminovaleric acid (5‐AVA), glutaric acid (GTA), 5‐hydroxyvaleric acid (5‐HV), and 1,5‐pentanediol (1,5‐PDO), have garnered significant attention due to their industrial applications. These chemicals are pivotal in synthesizing various high‐value products and materials, contributing to the versatility and applicability of biobased processes.^[^ ^4^, ^5^, ^6^, ^7^, ^8^, ^9^, ^10^, ^11^, ^12^, ^13^, ^14^, ^15^, ^16^, ^17^, ^18^, ^19^ ^]^

Accordingly, considerable attention has been directed toward the microbial production of 5‐HV and 1,5‐PDO due to their diverse applications (Figure S1, Supporting Information). Various metabolic pathways for 1,5‐PDO biosynthesis from glucose have been developed, employing different l‐lysine conversion modules such as the cadaverine‐derived pathway and the traditional DavBA‐mediated pathway. Additionally, distinct 5‐HV conversion modules have been explored, including the CoA‐dependent pathway and the CoA‐independent CAR‐based pathway. In an early study, an artificial 1,5‐PDO biosynthesis pathway was constructed using the DavBA‐mediated l‐lysine conversion module and CAR‐based 5‐HV conversion module, progressing through intermediates such as 5‐AVA, glutarate semialdehyde, 5‐HV, and 5‐hydroxyvaleraldehyde in recombinant *Escherichia coli*, resulting in 0.97 g L^−1^ 1,5‐PDO production.^[^ ^11^ ^]^ Another study introduced a CoA‐dependent 5‐HV conversion module in *E. coli*, where 5‐HV was converted to 1,5‐PDO via 5‐HV‐CoA and 5‐hydroxyvaleraldehyde through sequential enzymatic reactions involving CoA‐transferase, CoA‐acylating aldehyde dehydrogenase, and aldehyde reductase. This pathway achieved 3.19 g L^−1^ 5‐HV and 0.35 g L^−1^ 1,5‐PDO from glucose and l‐lysine.^[^ ^12^ ^]^ More recently, a cadaverine‐derived l‐lysine conversion module was employed to synthesize 1,5‐PDO. In this approach, l‐lysine was transformed into 5‐HV via intermediates such as cadaverine, 5‐aminovaleraldehyde, 5‐AVA, and glutarate semialdehyde. The subsequent conversion of 5‐HV into 1,5‐PDO was facilitated through a CAR‐mediated pathway. Using this design, an engineered *E. coli* strain harboring the pathway achieved a production titer of 9.25 g L^−1^ 1,5‐PDO.^[^ ^16^ ^]^

The microbial production of 1,5‐PDO is intricately linked to the l‐lysine‐derived pathway, highlighting the potential of *Corynebacterium glutamicum* as a robust host strain. Known for its high efficiency in synthesizing l‐lysine and other C5 chemicals, including 5‐AVA, GTA, and 5‐HV, *C. glutamicum* could serve as an ideal platform for developing 1,5‐PDO biosynthetic processes.^[^ ^5^, ^6^, ^7^, ^8^, ^9^, ^10^, ^13^, ^14^, ^15^, ^17^ ^]^ However, the bio‐based production of 1,5‐PDO presents significant technical challenges, primarily due to enzyme inefficiencies and energy demands across its biosynthetic pathways.^[^ ^12^, ^16^ ^]^ For 5‐AVA synthesis, two distinct pathways have been explored: the cadaverine‐based pathway and the traditional DavBA‐mediated pathway. The cadaverine‐based pathway is more energy‐ and electron‐efficient, generating fewer by‐products, recycling glutamate, and eliminating the need for molecular oxygen. These features make it advantageous for large‐scale anaerobic fermentation processes. However, in *C. glutamicum*, cadaverine is excreted as an end‐product or byproduct, thereby reducing overall pathway efficiency.^[^ ^20^, ^21^, ^22^ ^]^ In contrast, the DavBA‐mediated pathway, although less efficient in terms of energy and electron utilization, has been reported to achieve higher levels of 5‐AVA production in *C. glutamicum* while minimizing significant byproduct accumulation.^[^ ^5^, ^7^, ^10^, ^17^ ^]^ For the conversion of 5‐HV to 1,5‐PDO, two artificial modules have been developed for this conversion: the CoA‐dependent module and the CAR‐based direct reduction module. The CoA‐dependent pathway is theoretically more energy‐efficient, consuming less ATP per reaction. However, its practical utility is constrained by the low activity and substrate specificity of key enzymes such as CoA transferases and CoA‐acylating aldehyde dehydrogenases, resulting in low product titers. In contrast, the CAR‐based module benefits from a lower thermodynamic barrier and irreversible reductions, providing a robust driving force for 1,5‐PDO production. This makes the CAR‐based pathway a more industrially applicable route when enzyme inefficiencies hinder the CoA‐dependent route, countering its disadvantage of lower energy efficiency.

A comparative analysis of these modules reveals trade‐offs between energy efficiency and enzyme activity. The cadaverine‐based 5‐AVA module paired with the CoA‐dependent 1,5‐PDO module theoretically achieves the highest yield but is hampered by enzyme limitations.^[^ ^16^ ^]^ Alternatively, the DavBA‐mediated 5‐AVA pathway coupled with the CAR‐based 1,5‐PDO module provides a better balance of pathway efficiency and enzyme activity, making it more suitable for implementation in *C. glutamicum*. This approach was exemplified in an engineered *C. glutamicum* strain capable of producing 5‐HV, a key precursor for the subsequent conversion to 1,5‐PDO. In this 5‐HV biosynthetic pathway, the initial three steps of the l‐lysine catabolic pathway, mediated via 5‐aminovaleramide and encoded by the *Pseudomonas putida davTBA* genes, were followed by an intracellular reduction step catalyzed by the *E. coli yahK* gene. By integrating an artificial 5‐HV biosynthesis pathway and eliminating byproduct pathways via *gabD2* deletion, the engineered strain produced 52.1 g L^−1^ of 5‐HV during fed‐batch fermentation, achieving a yield of 0.33 g g^−1^ glucose.^[^ ^13^ ^]^ This result underscores the potential of *C. glutamicum* as a platform for efficient 1,5‐PDO biosynthesis.

**Figure 1:**
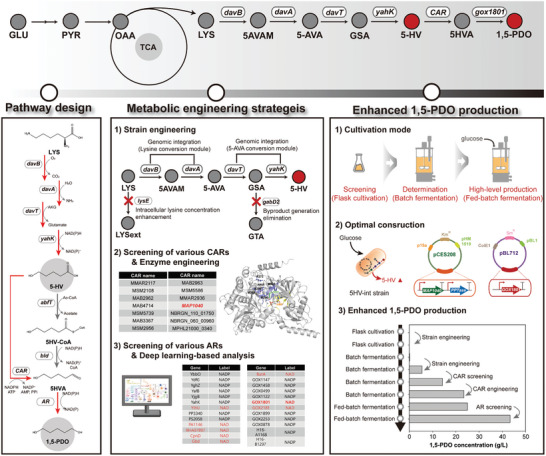
Schematic diagram of the metabolic engineering project for the development of 1,5‐PDO‐producing *C. glutamicum*. The abbreviations used are GLU, glucose; PYR, pyruvate; OAA, oxaloacetate; LYS, l‐lysine; 5‐AVAM, 5‐aminovaleramide; 5‐AVA, 5‐aminovaleric acid; GSA, glutarate semialdehyde; 5‐HV, 5‐hydroxyvaleric acid; 5HVA, 5‐aminovaleramide; and 1,5‐PDO, 1,5‐pentanediol.

Here, we address the metabolic engineering of *C. glutamicum* to produce 1,5‐PDO (**Figure** **1**). We first compared the CoA‐dependent and CoA‐independent 5‐HV conversion modules to establish an efficient biosynthetic pathway for 1,5‐PDO. Subsequent metabolic engineering of the base strain was conducted to optimize the production of the key precursor, 5‐HV, thereby enhancing the overall synthesis of 1,5‐PDO. Iterative improvements were then applied to increase the efficiency of each step in the 1,5‐PDO biosynthesis pathway. We systematically evaluated various carboxylic acid reductases (CARs) and their mutants using rational enzyme engineering techniques to identify the most suitable enzyme for converting 5‐HV into 5‐hydroxyvaleraldehyde. Next, we focused on identifying the most efficient aldehyde reductase for converting 5‐hydroxyvaleraldehyde into 1,5‐PDO. Ultimately, fed‐batch fermentation of the engineered strain produced 43.4 g L^−1^ 1,5‐PDO.

## 2 Results and Discussion

### 2.1 Construction of the 1,5‐PDO Biosynthesis System

**Figure 2:**
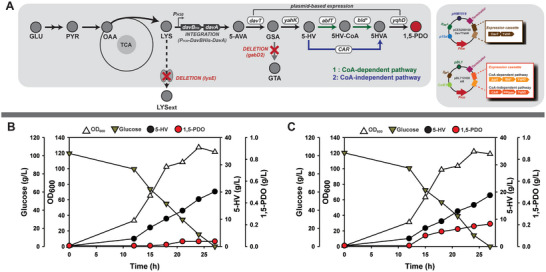
A) Metabolic pathway for the biosynthesis of 1,5‐PDO and plasmid construction for the expression of heterologous genes encoding the 1,5‐PDO biosynthesis pathway. The abbreviations used are GLU, glucose; PYR, pyruvate; OAA, oxaloacetate; LYS, l‐lysine; 5‐AVAM, 5‐aminovaleramide; 5‐AVA, 5‐aminovaleric acid; GSA, glutarate semialdehyde; 5‐HV, 5‐hydroxyvaleric acid; 5HV‐CoA, 5‐hydroxyvaleryl‐CoA; 5HVA, 5‐aminovaleramide; and 1,5‐PDO, 1,5‐pentanediol. B) Batch fermentation of the *C. glutamicum* 15PDO‐1 strain for 1,5‐PDO production. C) Batch fermentation of the *C. glutamicum* 15PDO‐2 strain for 1,5‐PDO production.

In our previous study, we identified the DavB‐DavA‐DavT‐YahK‐mediated pathway as the most promising 5‐HV biosynthesis pathway in *C. glutamicum*; the fed‐batch fermentation of *C. glutamicum* harboring this pathway, along with *gabD2* deletion, produced 52.1 g L^−1^ 5‐HV.^[^ ^13^ ^]^ Consequently, we investigated the further conversion of 5‐HV into 1,5‐PDO on the basis of the established 5‐HV biosynthesis pathway. The previously reported CoA‐dependent (*Clostridium aminobutyricum* AbfT + *Clostridium saccharoperbytulacetonicum* Bld^L273T^ + *E. coli* YqhD)^[^ ^12^ ^]^ and CoA‐independent (*Mycobacterium marinum* MMAR2117 + *Bacillus subtilis* PPTase + *E. coli* YqhD) ^[^ ^11^, ^16^ ^]^ pathways were examined via the engineered *C. glutamicum ΔgabD* (pCES208H30DavTYahKDavBhisA + pBL712H30AbfTBld^L273T^YqhD or pBL712H30MMAR2117PPTaseYqhD) strain through 120 h of flask cultivation. However, only l‐lysine, GTA, and 5‐HV were produced after cultivation, with no conversion to 1,5‐PDO (Figure S2, Supporting Information). Additionally, the transformation efficiency was too low, likely due to the large plasmid size. Therefore, based on the previous finding, the l‐lysine conversion module (P_H30_DavBhisA) was integrated into the chromosome of the *C. glutamicum* Δ*gabD* strain by disrupting *lysE* to prevent l‐lysine into the culture medium.^[^ ^13^, ^23^, ^24^, ^25^, ^26^, ^27^ ^]^ As a result, we engineered a recombinant strain, *C. glutamicum* Δ*gabD* Δ*lysE*::P_H30_DavBhisA (referred to as *C. glutamicum* AVA‐int‐gd). To verify the functionality of this integrated module, flask cultivations of the *C. glutamicum* AVA‐int‐gd strain were performed to assess its ability to produce 5‐AVA (Figure S3, Supporting Information). Next, for 5‐HV production, the plasmid‐based expression module of *davT* and *yahK* was introduced to 5‐AVA‐producing *C. glutamicum* strain AVA‐int‐gd. Further flask cultivation of *C. glutamicum* AVA‐int‐gd harboring pCES208H30DavTYahK was performed to assess its ability to produce 5‐HV (Figure S3, Supporting Information). After confirming that each strain could produce 5‐AVA and 5‐HV, we introduced downstream pathways for CoA‐dependent conversion (AbfT‐Bld^L273T^‐YqhD) and CoA‐independent conversion (MMAR2117‐PPTase‐YqhD), resulting in the development of the 15PDO‐1 and 15PDO‐2 strains, respectively (**Figure** **2**A). However, the flask cultivation of strains 15PD0‐1 and 15PDO‐2 did not yield 15PDO (Figure S4, Supporting Information). To assess pathway functionality, we performed further batch fermentations of the 15PDO‐1 (Figure 2B) and 15PDO‐2 (Figure 2C) strains. For the 15PDO‐1 strain, minimal 1,5‐PDO production (0.04 g L^−1^) was achieved, with a substantial amount of 5‐HV (20.16 g L^−1^) remaining without undergoing further conversion. For the batch fermentation of 15PDO‐2 harboring the CoA‐independent pathway, 0.20 g L^−1^ 1,5‐PDO and 18.79 g L^−1^ 5‐HV were produced. Although both strains produced only marginal levels of 1,5‐PDO, the pathways were confirmed to be functional in *C. glutamicum*, as in the case of *E. coli*. Among the two pathways, the CoA‐independent (CAR‐based) pathway was more effective for 1,5‐PDO generation in *C. glutamicum*. Consequently, further engineering efforts were undertaken on the basis of the 15PDO‐2 strain.

### 2.2 Improvement of the 5‐HV Production System in *C. glutamicum* and its Further Application to 1,5‐PDO Biosynthesis

**Figure 3:**
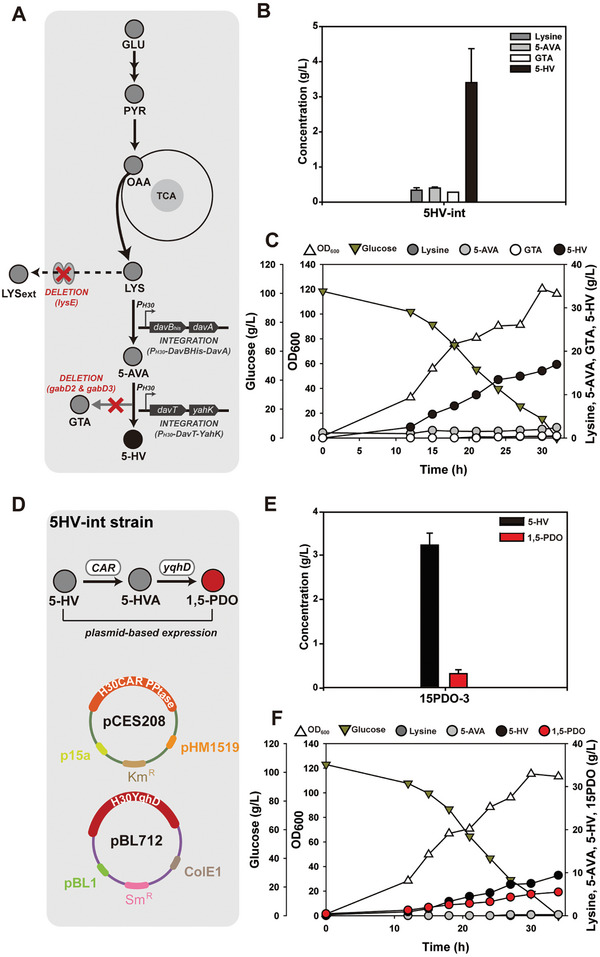
A) Metabolic engineering strategy for the development of the *C. glutamicum* 5HV‐int strain. The abbreviations used are GLU, glucose; PYR, pyruvate; OAA, oxaloacetate; LYS, l‐lysine; 5‐AVAM, 5‐aminovaleramide; 5‐AVA, 5‐aminovaleric acid; GSA, glutarate semialdehyde; 5‐HV, 5‐hydroxyvaleric acid; 5HVA, 5‐aminovaleramide; and 1,5‐PDO, 1,5‐pentanediol. B) Flask cultivation of the *C. glutamicum* 5HV‐int strain for 5‐HV production. All flask cultures were performed in triplicate. The measurements are presented as the means ± standard deviations. C) Batch fermentation of the *C. glutamicum* 5HV‐int strain for 5‐HV production. D) Metabolic engineering strategy for the development of the *C. glutamicum* 15PDO‐3 strain. The abbreviations used are 5‐HV, 5‐hydroxyvaleric acid; 5HVA, 5‐aminovaleramide; and 1,5‐PDO, 1,5‐pentanediol. E) Flask cultivation of the *C. glutamicum* 15PDO‐3 strain for 1,5‐PDO production. All flask cultures were performed in triplicate. The measurements are presented as the means ± standard deviations. F) Batch fermentation of the *C. glutamicum* 15PDO‐3 strain for 1,5‐PDO production.

The present study aims to achieve high 1,5‐PDO production. To this end, it is crucial to establish an efficient 5‐HV production system that supports substantial metabolic flux toward 1,5‐PDO. This significance arises from the critical role of 5‐HV as the key precursor in 1,5‐PDO synthesis. The initially constructed 1,5‐PDO biosynthesis system was inefficient and suffered from low transformation efficiency because the final plasmid size exceeded 10 kb. As a result, further engineering of the *C. glutamicum* AVA‐int‐gd strain was performed by integrating a 5‐AVA conversion module (P_H30_‐DavTYahK) at the *gabD3* site. Consequently, the *C. glutamicum* Δ*gabD2* Δ*lysE*::H30*davB_His_A* Δ*gabD3*::H30*davTyahK* strain (*C. glutamicum* 5HV‐int strain) was developed (**Figure** **3**A). To summarize, building on the previously developed 5HV‐7 strain,^[^ ^13^ ^]^ which features the deletion of the *gabD2* gene and the introduction of plasmids pCES208H30DavTBhisA and pBL712H30YahK, additional engineering strategies were applied to further enhance 5‐HV production in *C. glutamicum*. These modifications included the deletion of the *lysE* gene, achieved through the integration of P_H30_‐DavBhisA, to block L‐lysine excretion and enhance L‐lysine conversion, as well as the deletion of the *gabD3* gene, facilitated by the integration of P_H30_‐DavTYahK, to prevent GTA formation and promote the conversion of 5‐AVA. Subsequent flask cultivation of the strain without plasmid‐based expression of the 5‐HV biosynthesis module produced 3.4 ± 0.97 g L^−1^ 5‐HV, which was 5.4 fold greater than that achieved with the 5HV‐7 strain.^[^ ^13^ ^]^ Additionally, there was a significant reduction in intermediate metabolites: 0.34 g L^−1^ ± 0.007 l‐lysine, 0.40 ± 0.03 g L^−1^ 5‐AVA, and 0.28 ± 0.001 g L^−1^ GTA (Figure 3B). Further batch fermentation of the 5HV‐int strain produced 16.93 g L^−1^ 5‐HV. Other metabolites were present at only minimal concentrations: 0.38 g L^−1^ l‐lysine, 0.67 g L^−1^ 5‐AVA, and 0.48 g L^−1^ GTA (Figure 3C). As a result, it is revealed that integrating P_H30_‐DavBhisA and P_H30_‐DavTYahK into the genome enabled pathways for a more stable and balanced expression. These targeted modifications significantly improved the efficiency of 5‐HV production, underscoring the effectiveness of precise genetic engineering in optimizing metabolic pathways and minimizing byproduct accumulation.

Next, the CAR‐based 5‐HV conversion module (MMAR2117‐PPTase‐YqhD), which achieved higher 1,5‐PDO production than the CoA‐based 5‐HV conversion module, was introduced into the *C. glutamicum* 5HV‐int strain (Figure 3D). The resulting strain, designated 15PDO‐3, was then examined through flask cultivation, yielding 3.2 ± 0.27 g L^−1^ 5‐HV and 0.3 ± 0.09 g L^−1^ 1,5‐PDO (Figure 3E), achieving the undetectable production of other metabolites. Further batch fermentation of the 15PDO‐3 strain was conducted to monitor cell growth and metabolite production over time (Figure 3F), producing 9.37 g L^−1^ 5‐HV and 5.48 g L^−1^ 1,5‐PDO. Negligible quantities of l‐lysine and 5‐AVA were detected, with no GTA accumulation observed. However, despite the successful production of 1,5‐PDO, a substantial amount of 5‐HV remained in the culture medium without further conversion to 1,5‐PDO. As a result, modification of the 1,5‐PDO conversion system was deemed necessary.

### 2.3 Examination of Different CAR Candidates to Improve the 1,5‐PDO Biosynthesis System

**Figure 4:**
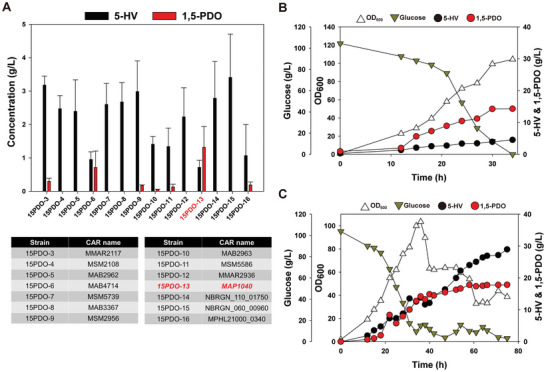
A) Flask cultivation of *C. glutamicum* 15PDO‐3 to 16 strain strains for 1,5‐PDO production. All flask cultures were performed in triplicate. The measurements are presented as the means ± standard deviations. B) Batch fermentation of the *C. glutamicum* 15PDO‐13 strain for 1,5‐PDO production. C) Fed‐batch fermentation of the *C. glutamicum* 15PDO‐13 strain for 1,5‐PDO production.

It was confirmed that 1,5‐PDO can be successfully produced in the established system. However, a low production level of 1,5‐PDO was observed, alongside inefficient conversion of 5‐HV into 1,5‐PDO. To address the issue of the inefficient conversion rate of 5‐HV into 1,5‐PDO, efforts were directed toward optimizing the reaction module mediated by CAR, which converts 5‐HV into 5‐hydroxyvaleraldehyde. Consequently, 13 additional CARs from various microorganisms were tested (Table S1, Supporting Information). These 13 different CARs were introduced into the 5HV‐int strain, along with the expression of PPTase and YqhD, resulting in the development of 13 distinct engineered *C. glutamicum* strains: *C. glutamicum* 15PDO‐4 to 15PDO‐16 (**Figure** **4**A). Further cultivation of the strains in flasks revealed that the conversion of 5‐HV into 1,5‐PDO by six out of the 13 CARs was effective. Among the six CARs, MAP1040 emerged as the most effective candidate for 1,5‐PDO production. The *C. glutamicum* 15PDO‐13 strain expressing MAP1040, PPTase, and YqhD produced 1.3 ± 0.62 g L^−1^ 1,5‐PDO and 0.72 ± 0.21 g L^−1^ 5‐HV. With the established *C. glutamicum* 15PDO‐13 strain, which achieved the highest 1,5‐PDO production in flask cultivation, further batch fermentation was conducted to examine whether MAP1040 could increase 1,5‐PDO production. This fermentation process produced 4.53 g L^−1^ 5‐HV and 14.30 g L^−1^ 1,5‐PDO (Figure 4B). Compared with that of the *C. glutamicum* 15PDO‐3 strain, 5‐HV accumulation decreased 0.27 fold, whereas 1,5‐PDO production increased 2.6 fold. This suggests that the newly introduced MAP1040 successfully converted 5‐HV to 1,5‐PDO via 5‐valeraldehyde. Moreover, other byproducts, such as l‐lysine, 5‐AVA, and GTA, were not detected.

Subsequently, fed‐batch fermentation of the *C. glutamicum* 15PDO‐13 strain was performed to increase 1,5‐PDO production (Figure 4C). However, the fed‐batch fermentation results exhibited distinct patterns of cell growth reduction compared with those of previous *C. glutamicum* fed‐batch fermentations.^[^ ^9^, ^13^, ^14^, ^20^, ^28^ ^]^ During fermentation, a notable reduction in cell growth was observed after 36 h, with the OD_600_ decreasing from a peak of 103.52–89.72. This decline continued throughout fermentation, resulting in a final OD_600_ of 38.71 after 75 h. Despite the consistent glucose feeding and the maintenance of its concentration at 10–20 g L^−1^, there was no substantial increase in 1,5‐PDO production. By the 48th h of fed‐batch fermentation, the production of 1,5‐PDO and 5‐HV reached 16.33 and 17.01 g L^−1^, respectively, with a productivity rate of 0.34 g·L^−1^·h^−1^. After 72 h of fermentation, the final concentration of 1,5‐PDO reached 17.83 g L^−1^, while that of 5‐HV increased significantly, peaking at 28.93 g L^−1^. The total carbon yield of the metabolites was calculated as 0.32 mol mol^−1^. These issues, including a sharp decline in cell growth and inefficient conversion of 5‐HV to 1,5‐PDO in the later stages of fed‐batch fermentation, highlight the need for further modifications to the established system. Therefore, additional exploration and adjustments were deemed necessary to increase 1,5‐PDO production.

### 2.4 Engineering a Carboxyl Group Reduction Module to Increase 1,5‐PDO Production

The accumulation of 5‐HV in the culture medium indicated that the conversion of 5‐HV into 1,5‐PDO via 5‐hydroxyvaleraldehyde was a limiting step. This phenomenon is presumably due to 5‐hydroxyvaleraldehyde, a highly reactive intermediate formed from 5‐HV, which poses challenges in the 1,5‐PDO biosynthesis pathway because of its potential to interact with and modify enzyme structures. Aldehydes are highly electrophilic compounds known to exhibit reactivity with various biomolecules, including amino acids and cellular membranes. For example, glycolaldehyde has been reported to interact with the amino and thiol groups of amino acids, inducing protein cross‐linking and compromising structural stability.^[^ ^29^ ^]^ Similarly, long‐chain alkanals such as hexaldehyde and 4‐hydroxynonanal have been shown to react with cell membranes, causing damage, and to modify proteins through interactions with lysine, cysteine, and histidine residues. These reactions often involve the formation of Schiff bases, resulting in structural modifications and functional inactivation of proteins. In the case of 5‐hydroxyvaleraldehyde, similar toxic effects are hypothesized due to its reactivity with cell membranes and specific amino acids within enzymes. To investigate these potential interactions, structural modeling and computational analysis using MoleOnline were performed to identify the aldehyde product release pathway in the R‐domain of the MAP1040. The analysis revealed several residues along the putative product pathway that are prone to aldehyde reactivity, including three lysines, one cysteine, and four histidines (Figure S5, Supporting Information). These residues are highly susceptible to Schiff base formation and other covalent modifications, which could lead to structural destabilization or inactivation of the enzyme. This finding highlights the inherent challenge of aldehyde accumulation during bioproduction processes. The reactivity of 5‐hydroxyvaleraldehyde could contribute to both enzyme inactivation and cell viability reduction. Therefore, optimizing the reaction rate of 5‐hydroxyvaleraldehyde and its subsequent conversion to 1,5‐PDO is critical for maximizing the 1,5‐PDO productivity.

**Figure 5:**
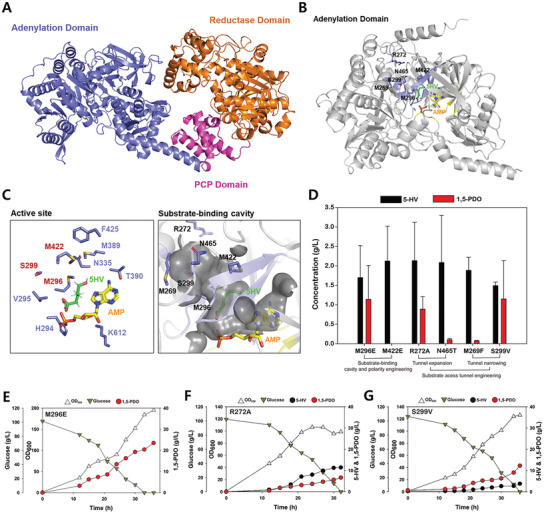
A) Structure of carboxylic acid reductase (MAP1040) modeled by AlphaFold2, which consists of three multiple domains. B) Mutation sites within the adenylation domain of the CAR. C) Close‐up view of the active site and substrate‐binding cavity. The substrate 5‐HV is colored in green, and AMP is colored in yellow. D) Metabolic engineering strategies devised for the development of efficient CAR mutants: flask cultivation of *C. glutamicum* 15PDO strains for 1,5‐PDO production. All flask cultures were performed in triplicate. The measurements are presented as the means ± standard deviations. E) Batch fermentation of the *C. glutamicum* 15PDO‐13(M296E) strain for 1,5‐PDO production. F) Batch fermentation of the *C. glutamicum* 15PDO‐13(R272A) strain for 1,5‐PDO production. G) Batch fermentation of the *C. glutamicum* 15PDO‐13(S299V) strain for 1,5‐PDO production.

In this regard, the catalytic activity of CAR (MAP1040) was fine‐tuned by modulating its catalytic efficiency. The catalytic efficiency of MAP1040 was altered by engineering the amino acid residues near the active site and substrate‐binding cavity of the enzymes. For example, S299 was replaced with a bulkier amino acid residue (valine) to reduce the size of the substrate‐binding cavity. Additionally, mutations, such as M296E, M422E, R272A, N465T, and M269F, were introduced to alter the size and polarity of the active site and substrate‐binding cavity (**Figure** **5**A,B,C). After each mutation was introduced into the enzyme, the resulting plasmids, pCES208H30MAP1040mutPPTase and pBL712H30YqhD, were transformed into the 5HV‐int strain, generating the 15PDO‐13(mutant) strains. Then, flask cultivation was performed for each strain (Figure 5D). The introduction of the M296E, R272A, and S299V mutants resulted in 1,5‐PDO production levels comparable to those of wild‐type MAP1040. Specifically, the 15PDO‐13(M296E) strain produced 1.7 ± 0.82 g L^−1^ 5‐HV and 1.14 ± 0.87 g L^−1^ 1,5‐PDO, the 15PDO‐13(R272A) strain generated 2.14 ± 0.98 g L^−1^ 5‐HV and 0.89 ± 0.32 g L^−1^ 1,5‐PDO, and the 15PDO‐13(S299V) strain yielded 1.49 ± 0.09 g L^−1^ 5‐HV and 1.15 ± 0.98 g L^−1^ 1,5‐PDO. However, the other recombinant strains presented lower 1,5‐PDO concentrations. Therefore, batch fermentations of the 15PDO‐13(M296E; Figure 5E), 15PDO‐13(R272A; Figure 5f), and 15PDO‐13(S299V; Figure 5G) strains were conducted to examine the effects of CAR activity variations on 1,5‐PDO productivity in detail.

Remarkably, the 15PDO‐13(M296E) strain demonstrated a significant increase in 1,5‐PDO production, reaching a peak concentration of 23.5 g L^−1^ without the formation of byproducts, such as l‐lysine, 5‐AVA, GTA, and 5‐HV (Figure 5E). This strain also achieved a peak OD_600_ of 195.1, approximately double that of the 15PDO‐13 strain. In contrast, the other strains exhibited similar or inferior 1,5‐PDO production compared with the 15PDO‐13 strain. This result indicates that the intracellular catalytic activity of the MAP1040_M296E variant is nearly optimal for converting 5‐HV into 1,5‐PDO via 5‐hydroxyvaleraldehyde. Then, on the basis of the favorable M296E mutant, (M296E/S299V) and (M296E/R272A) double mutants were generated to explore potential synergistic effects. However, subsequent batch fermentation of these strains did not improve 1,5‐PDO production (Figure S6, Supporting Information).

**Figure 6:**
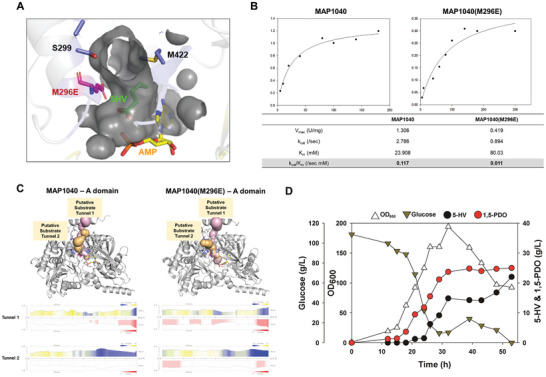
A) Close‐up view of the substrate‐binding cavity of the M296E mutant protein. The substrate 5‐HV is colored in green, and AMP is colored in yellow. B) Kinetic parameters of MAP1040 and its mutant (M296E). C) Substrate tunnel analysis of MAP1040 and its mutant (M296E). D) Fed‐batch fermentation of the *C. glutamicum* 15PDO‐13(M296E) strain for 1,5‐PDO production.

To understand the high product yield achieved during batch fermentation of the 15PDO‐13(M296E) strain, we performed a detailed analysis of the substrate‐binding cavity in the MAP1040(M296E) model (**Figure** **6**A) and conducted kinetic studies of both MAP1040 and its variant (Figure 6B). Kinetic analysis, based on NADPH oxidation, revealed that the catalytic efficiency of the MAP1040(M296E) enzyme was 11 fold lower than that of the wild‐type enzyme (Figure 6B). This finding suggests that the M296E mutation reduced the rate of 5‐hydroxyvaleraldehyde formation, likely preventing aldehyde accumulation within recombinant cells. Such a reduction in aldehyde levels may alleviate stress on both enzymes and cells.

Comparative analysis of the substrate tunnels in the wild‐type MAP1040 and M296E mutant revealed significant changes in polarity. Specifically, the substitution of methionine with glutamate at position 296 introduced a pronounced negative electrostatic potential to the substrate tunnel. This alteration is hypothesized to cause electrostatic repulsion with the carboxylate (COO⁻) group of 5‐HV, likely hindering the efficient formation of the ES complex in the active site (Figure 6C). This corresponds to the previous steady‐state kinetic results, where the K_m_ value of M296E was significantly lower than that of the wild‐type. This effect may explain the higher cell density observed during fermentation, as reduced aldehyde accumulation would alleviate cellular toxicity. Overall, these results suggest that maintaining CAR activity at a level that prevents accumulation of the toxic reaction intermediate, 5‐hydroxyvaleraldehyde, is crucial. Based on these findings, the 15PDO‐13(M296E) strain, which demonstrated the highest 1,5‐PDO production, was selected for further optimization and investigation through fed‐batch fermentation.

During fed‐batch fermentation of the 15PDO‐13(M296E) strain, the glucose concentration was carefully maintained between 10 and 20 g L^−1^ (Figure 6D). At the end of fermentation, the final concentrations of 1,5‐PDO and 5‐HV were 24.99 and 22.01 g L^−1^, respectively, with no other byproducts detected. However, similar to the 15PDO‐13 strain, there was no significant improvement in 1,5‐PDO production despite continuous glucose feeding and maintenance within the specified concentration range. Notably, a substantial decrease in cell growth was observed after 39 h, mirroring the pattern observed in the fed‐batch fermentation of the 15PDO‐13 strain. After 39 h of fed‐batch fermentation, 1,5‐PDO production reached 21.39 g L^−1^, with a yield of 0.12 mol mol^−1^ and a productivity of 0.55 g·L^−1^·h^−1^. Concurrently, 14.34 g L^−1^ 5‐HV was produced, and the total carbon yield of the l‐lysine‐derived metabolites was 0.19 mol mol^−1^. Beyond this point, no further increase in 1,5‐PDO production was observed, with the final titer reaching 19.35 g L^−1^ 1,5‐PDO. On the other hand, 5‐HV production increased, reaching its highest titer of 22.01 g L^−1^ at the end of fermentation. While the overarching trends in the results mirrored those obtained from the fed‐batch fermentation of the 15PDO‐13 strain, fed‐batch fermentation of the 15PDO‐13(M296E) strain revealed a distinct outcome, with rapid cell growth, glucose consumption, and concurrent 1,5‐PDO production. This accelerated fermentation demonstrated that the MAP1040(M296E) mutant played a pivotal role in enhancing 1,5‐PDO production. However, the limited increase in 1,5‐PDO production rate despite continuous glucose feeding and the excess 5‐HV accumulation strongly suggest potential bottlenecks or limitations within the engineered pathway.

### 2.5 Engineering an Aldehyde Group Reduction Module to Achieve Elevated Levels of 1,5‐PDO Production

It was found that both 15PDO‐13 and 15PDO‐13(M296E) strains exhibited limited increases in 1,5‐PDO production and a sharp decline in cell growth after the peak OD_600_ was reached during the fed‐batch fermentations. Therefore, we hypothesized that the toxic aldehyde intermediate 5‐hydroxyvaleraldehyde might be responsible for inhibiting cell growth. In previous fed‐batch fermentations, continuous accumulation of 5‐HV was observed. This observation suggests that 5‐hydroxyvaleraldehyde, which may cause cellular toxicity, is preferentially oxidized to 5‐HV rather than reduced to 1,5‐PDO, a process that relies on NAD(P)H as a cofactor. To evaluate the hypothesis, the 15PDO‐13(M296E)‐X strain was developed by introducing MAP1040(M296E) and PPTase into the 5HV‐int strain, with *yqhD* expression omitted. In this setup, the final step in 1,5‐PDO production—the reduction of 5‐hydroxyvaleraldehyde to 1,5‐PDO—relied on the action of *yahK*, which was integrated into the genome of the 5HV‐int strain. Batch fermentation of the 15PDO‐13(M296E)‐X strain was conducted to examine the patterns of cell growth and metabolite production. Compared with the 15PDO‐13(M296E) strain expressing MAP1040(M296E), PPTase, and YqhD, the 15PDO‐13(M296E)‐X strain expressing MAP1040(M296E) and PPTase exhibited a decrease in both the maximum OD_600_ (114.29) and 1,5‐PDO production (8.68 g L^−1^) (Figure S7, Supporting Information). This outcome supports the hypothesis that 5‐hydroxyvaleraldehyde is toxic to the cell, leading to reduced cell growth. To alleviate this toxicity, the oxidation of 5‐hydroxyvaleraldehyde to 5‐HV may be the preferred approach. In light of these findings, the *yqhD* gene was expressed on the basis of a high‐copy‐number plasmid, pHCP.^[^ ^30^ ^]^

To examine whether an increase in the gene copy number would increase 1,5‐PDO production, the modified high‐copy plasmid pHCPH30‐MCS was used to express the *yqhD* gene. Next, two plasmids, pHCPH30YqhD and pBL712H30MAP1040(M296E)PPTaseYqhD, were cotransformed into the *C. glutamicum* 5HV‐int strain, generating the *C. glutamicum* 15PDO‐13(M296E)‐H strain. Batch fermentation of this strain was then conducted. However, unlike the expectation that high‐copy number plasmids would lead to elevated gene expression and increased product yield, the batch fermentation results did not show an increased production rate (Figure S8, Supporting Information), achieving 1,5‐PDO production of 3.54 g L^−1^ reached its highest titer at the end of fermentation. In contrast, 5‐HV accumulated at a high concentration of 14.24 g L^−1^. The discrepancy between the expected and actual outcomes may arise from the high expression level of *yqhD*, which catalyzes both the reduction of glutarate semialdehyde to 5‐HV and the reduction of 5‐hydroxyvaleraldehyde to 1,5‐PDO, with NADPH serving as a cofactor. In the 1,5‐PDO biosynthetic pathway we constructed, the production of 1 mole of 1,5‐PDO requires a total of 7 moles of NADPH and 1 mole of ATP. This includes 4 moles of NADPH for l‐lysine biosynthesis and an additional 3 moles of NADPH along with 1 mole of ATP for the conversion of l‐lysine to 1,5‐PDO. Given the heavy reliance of the established metabolic pathway on NADPH, increasing the action of *yqhD* would further strain the available NADPH supply. Therefore, rather than amplifying *yqhD* expression, which intensifies NADPH consumption, a more balanced reaction step needs to be devised. This may involve enhancing NADPH regeneration systems or identifying alternative enzymes that function more efficiently under existing metabolic conditions.

To address the NADPH limitation in 1,5‐PDO biosynthesis, several strategies were employed to enhance NADPH availability. These included introducing transhydrogenases, PntAB and UdhA from *E. coli*, which transfer electrons from NADH to NADP⁺; NADH kinases, Pos5 from *S. cerevisiae*, which phosphorylate NADH to generate NADPH; and glucose dehydrogenases, GDH from *B. subtilis*, which regenerate NADPH from NADP⁺.^[^ ^19^, ^31^, ^32^, ^33^ ^]^ Accordingly, these genes were incorporated into the *C. glutamicum* 15PDO‐13(M296E) strain, resulting in derivative strains: 15PDO‐13(M296E)ecp, 15PDO‐13(M296E)ecu, 15PDO‐13(M296E)scp, and 15PDO‐13(M296E)bsg (Figure S9a, Supporting Information). However, flask cultivations of the engineered strains adversely showed a significant decrease in 1,5‐PDO production (Figure S9b, Supporting Information). These results indicate that enhancing NADPH regeneration alone is insufficient to improve 1,5‐PDO production and may even have detrimental effects. This limitation is likely due to a combination of factors, including metabolic imbalances, competition for cellular resources such as ATP, and unanticipated interactions within the metabolic network. Furthermore, these findings highlight the challenges of static NADPH regulation strategies, which cannot adapt to the dynamic NADPH demands that occur during different phases of cell growth and production.^[^ ^34^ ^]^ In the case of 1,5‐PDO biosynthesis, which imposes a high NADPH demand, such rigid approaches often result in imbalances in the NADPH/NADP⁺ ratio, leading to disruptions in cell growth and production efficiency. This underscores the importance of developing dynamic, context‐specific strategies for NADPH regulation to effectively balance redox homeostasis and meet the metabolic demands of high‐performance bioproduction systems.^[^ ^34^ ^]^

**Figure 7:**
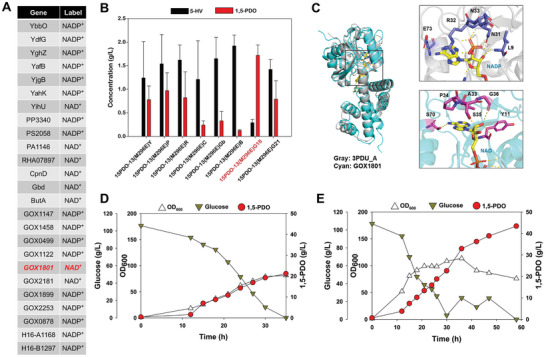
A) Deep learning‐based analysis of various aldehyde reductases. B) Flask cultivation of *C. glutamicum* strains for 1,5‐PDO production. All flask cultures were performed in triplicate. The measurements are presented as the means ± standard deviations. C) Structural analysis and cofactor docking simulation of GOX1801 in comparison with γ‐hydroxybutyrate dehydrogenase from *G. sulfurreducens* (PDB: 3PDU). D) Batch fermentation of the *C. glutamicum* 15PDO‐13(M296E)G18 strain for 1,5‐PDO production. E) Fed‐batch fermentation of the *C. glutamicum* 15PDO‐13(M296E)G18 strain for 1,5‐PDO production.

Subsequently, various aldehyde reductases from different microorganisms which utilized NADH as a cofactor were assessed to tackle the heavy reliance on NADPH of *yqhD*.^[^ ^35^, ^36^, ^37^ ^]^ Accordingly, the cofactor preference prediction for aldehyde reductase candidates was performed using the deep learning‐based DISCODE model. This model was pre‐trained on a diverse dataset of 7,132 NAD(P)^+^‐binding sequences from the Swiss‐Prot database, encompassing a wide range of structural domains and ensuring the universality of its predictions.^[^ ^38^ ^]^ Using the deep learning‐based DISCODE model, the cofactor preference for NAD(P)^+^ was evaluated for 25 aldehyde reductases from various microorganisms on the basis of previous reports and the KEGG database. Among them, eight aldehyde reductases showed a preference for NADH (Table S2, Supporting Information; **Figure** **7**A). These eight aldehyde reductases (YihU, PA1146, RHA07897, CpnD, Gbd, ButA, GOX1801, and GOX2181) were then tested for 1,5‐PDO production through flask cultivation, replacing the *yqhD* gene. Consequently, *C. glutamicum* strains 15PDO‐13(M296E)Y, 15PDO‐13(M296E)P, 15PDO‐13(M296E)R, 15PDO‐13(M296E)C, 15PDO‐13(M296E)Gb, 15PDO‐13(M296E)B, 15PDO‐13(M296E)G18, and 15PDO‐13(M296E)G21 were developed by expressing MAP1040(M296E), PPTase, and each aldehyde reductase (YihU, PA1146, RHA07897, CpnD, Gbd, ButA, GOX1801, and GOX2181, respectively). After flask cultivation of each strain, GOX1801 was identified as the most effective aldehyde reductase for the final reduction of 5‐hydroxyvaleraldehyde to 1,5‐PDO, supported by the highest 1,5‐PDO production, with a final titer of 1.72 ± 0.22 g L^−1^ (Figure 7B). A structure‐based computational analysis was performed to examine the cofactor binding site of GOX1801 (Figure 7C). In typical NADP^+^‐dependent enzymes, such as γ‐hydroxybutyrate dehydrogenase from *Geobacter sulfurreducens* (PDB: 3PDU), residues like N31 and R32 are conserved to interact with the phosphate group of NADPH.^[^ ^39^ ^]^ In contrast, GOX1801 was found to possess alanine and proline at these positions, which likely limits its ability to effectively interact with phosphate. Instead, S35 is proposed to form hydrogen bonds with the hydroxyl groups of NAD^+^, thereby facilitating its stable association within the binding site.

Next, batch fermentation of the strain was performed (Figure 7D). As a result, the *C. glutamicum* 15PDO‐13(M296E)G18 strain could produce comparable amounts of 1,5‐PDO compared with the *C. glutamicum* 15PDO‐13(M296E) strain, with a final titer of 21.2 g L^−1^ and no formation of byproducts. Although the maximum OD_600_ was lower than that of *C. glutamicum* 15PDO‐13(M296E), we proceeded with fed‐batch fermentation to verify whether the strain could sustain continuous 1,5‐PDO production while maintaining its biomass. During fed‐batch fermentation of the 15PDO‐13(M296E)G18 strain, the glucose concentration was maintained within a range of 10–20 g L^−1^ (Figure 7E). Unlike the previous fed‐batch fermentation patterns, the strain maintained its biomass at an OD_600_ of ≈100 for 20 h, after which it gradually decreased. Furthermore, 1,5‐PDO production continued to increase, reaching a peak of 43.4 g L^−1^ with a yield of 0.48 mol mol^−1^ without any other by‐products detected. This represents the highest reported titer to date. This accomplishment highlights the success of our metabolic engineering strategy and highlights the potential of *C. glutamicum* as a viable platform for the industrial bioproduction of valuable chemicals, such as 1,5‐PDO.

## 3 Conclusion

In this study, we successfully constructed and optimized a 1,5‐PDO biosynthesis system in *C. glutamicum*. Our initial approach incorporated both CoA‐dependent and CoA‐independent pathways for converting 5‐HV to 1,5‐PDO. Notably, the CoA‐independent pathway, which utilizes the CAR enzyme, was more effective in *C. glutamicum*. Subsequent efforts focused on enhancing the production of 5‐HV, a key intermediate in 1,5‐PDO biosynthesis. Through targeted genetic modifications and strain engineering, we developed the 5HV‐int strain, which significantly improved 5‐HV production while minimizing the accumulation of unwanted byproducts. Through this process, the introduction of the optimized CoA‐independent conversion module into the strain resulted in the creation of the 15PDO‐3 strain, which exhibited a marked increase in 1,5‐PDO production. Despite these advancements, the incomplete conversion of 5‐HV to 1,5‐PDO, which results in 5‐HV accumulation, was observed. To address this issue, we comprehensively screened different CAR enzyme candidates, ultimately identifying MAP1040 as the most effective enzyme for converting 5‐HV to 1,5‐PDO. Further optimization of MAP1040 through strategies such as substrate‐binding cavity engineering, polarity modulation, and substrate access tunnel engineering resulted in the M296E mutant, which significantly enhanced 1,5‐PDO production. Nevertheless, the pursuit of efficient 1,5‐PDO production revealed ongoing challenges during fed‐batch fermentation, particularly in achieving substantial titer improvements. A deeper investigation into the metabolic constraints suggested a potential scarcity of NADPH, which is heavily utilized in both the l‐lysine and 1,5‐PDO biosynthesis pathways. To overcome this, we employed deep learning‐based analysis to screen various aldehyde reductase candidates and assess their preference for NADH or NADPH as reducing agents. Among the 25 candidates screened, eight demonstrated a preference for NADH, with the GOX1801 gene emerging as the most promising. The incorporation of GOX1801 resulted in a final 1,5‐PDO titer of 43.4 g L^−1^, underscoring its effectiveness in improving production. These iterative metabolic engineering efforts highlight the potential for developing an efficient and robust bioprocess for 1,5‐PDO production. Our findings provide valuable insights into the optimization of biosynthetic pathways and emphasize the importance of enzyme engineering, cofactor balancing, and strain development in advancing industrial biotechnology.

## 4 Experimental Section

### Materials and Strains

The chemicals used in this study were sourced from Tokyo Chemical Industry (TCI). A complete list of the bacterial strains and plasmids used can be found in Table S3 (Supporting Information). For general gene cloning, *Escherichia coli* XL1‐Blue (Stratagene, La Jolla, CA, USA) was used, with all DNA manipulations carried out according to standard molecular biology protocols.^[^ ^40^ ^]^

### Construction of Plasmids

The primers used in this study are detailed in Table S4 (Supporting Information) and were synthesized by Cosmogenetech (Seoul, Korea). The detailed procedures for plasmid construction are provided in Section S1 (Supporting Information). All CAR and PPTase genes^[^ ^41^, ^42^ ^]^ used in this study were kindly provided by Prof. Alexander F. Yakunin.

### *C*. *glutamicum* Genome Manipulation

Chromosomal gene deletion and integration of the gene expression cassette were carried out using homologous recombination via the plasmid pK19mobSacB.^[^ ^13^, ^20^ ^]^ Transformations using *C. glutamicum*–*E. coli* shuttle vectors were carried out via electroporation, followed by heat shock treatment.^[^ ^43^ ^]^ Full descriptions of the construction methods for each recombinant *C. glutamicum* strain used in this research can be found in Section S2 (Supporting Information).

### Culture Conditions

For DNA manipulation, *E. coli* XL1‐Blue was grown in Luria–Bertani medium (Difco™) supplemented with kanamycin (Km, 30 mg/L) and spectinomycin (Sp, 40 mg/L), depending on the resistance markers of the plasmids used.

The cultural conditions were outlined in previous research.^[^ ^13^, ^20^, ^44^ ^]^ To cultivate recombinant *C. glutamicum* strains for 5‐HV and 1,5‐PDO production, CG50 medium was utilized in flask cultures,^[^ ^13^, ^42^ ^]^ while seed cultures were prepared in RG medium.^[^ ^44^ ^]^ Batch and fed‐batch fermentations were carried out in a 5‐L fermenter (BioCNS, Korea) in which 1.5 L of CG100 medium was used under culture conditions of 30 °C and 600 rpm.^[^ ^13^, ^20^ ^]^ Km and Sp were added at final concentrations of 20 mg/L and 200 mg/L, respectively.

### Analytical Procedures

The methods used for analyzing cell growth and metabolites followed previously established protocols as detailed in the literature.^[^ ^13^ ^]^ Cell growth was specifically tracked by measuring the optical density at 600 nm (OD_600_), while metabolite concentrations were quantified via high‐performance liquid chromatography (HPLC). Additional details on sample preparation and analytical conditions can be found in the cited studies.^[^ ^13^, ^45^ ^]^

### Enzymatic Assays

For CAR kinetic studies, MAP1040 was cloned and expressed in recombinant *E. coli*. In addition, PPTase from *Bacillus subtilis* was coexpressed with MAP1040 to achieve maximum activity. Subsequently, MAP1040 was purified exclusively via Ni^+^ affinity chromatography. MAP1040 variants were generated through site‐directed mutagenesis and purified using the same methodology.

The steady‐state kinetics of MAP1040 were determined via an NADPH oxidation‐based assay at 340 nm and 30 °C via a spectrophotometer (Thermo Fisher Scientific, MA, USA). The reaction was conducted in a 1 mL mixture containing 100 mM HEPES buffer (pH 7.5), 10 mM MgCl_2_, 0.5 mM ATP, 0.1 mM NADPH, substrate (5–200 mM 5‐HV), and 50–100 µg of purified CAR enzymes.

### Substrate Docking Simulation

The 3D models of MAP1040 were generated using Alphafold2.^[^ ^46^ ^]^ In addition, cofactors (e.g., AMP and NADPH) were docked using AlphaFill.^[^ ^47^ ^]^ Considering that CARs are multi‐domain proteins, each domain (e.g., A‐domain and PCP‐R didomain) was isolated for ligand docking simulation. The AMP‐merged MAP1040 A‐domain was refined via the Schrödinger program, specifically the Protein Preparation Wizard, Epik, Prime, and OPLS3e force fields.^[^ ^48^, ^49^ ^]^ Subsequently, 5‐HV was docked into the substrate‐binding pocket via Glide SP and visualized with PyMOL. Additionally, substrate access tunnels were analyzed via MoleOnline.^[^ ^50^ ^]^

### Deep Learning‐Based NAD(P)H Cofactor Preference Identification

The DISCODE model (https://github.com/SBML‐Kimlab/DISCODE),^[^ ^38^ ^]^ which uses a deep learning architecture based on the Transformer encoder, was employed to determine the NAD(P)H cofactor preference of 25 collected aldehyde reductases. This model accepted an amino acid sequence as input, processed the embeddings with the ESM2 model, and then transferred the resulting embedding vectors to DISCODE, which calculated the probability of each NAD(P)H cofactor preference. To validate the deep learning‐based prediction, the cofactor docking simulation of GOX1801 was performed using an identical methodology.

## Conflict of Interest

The authors declare no conflict of interest.

## Author Contributions

S.J.P., S.Y.L., J.C.J., and J.B.P conceived the project. Y.J.S., S.‐Y.H., J.C.J., K.J.J., S.Y.L., J.‐B.P., and S.J.P designed the research. Y.J.S., S.‐Y.H., and H.Y.L. performed the experiments. J.K. and D.K. contributed materials/analysis tools. S.J. and J.Y.P. analyzed the data. Y.J.S., S.‐Y.H., J.C.J., J.K., D.K., K.J.J., S.Y.L., J.‐B.P., and S.J.P wrote the paper. All the authors have read and approved the final manuscript.

## Supporting information



Supporting Information

## Data Availability

The data that support the findings of this study are available from the corresponding author upon reasonable request
