# Supplementary material for: Metabolic Engineering of Corynebacterium glutamicum for High‐Level Production of 1,5‐Pentanediol, a C5 Diol Platform Chemical
Source: Adv Sci (Weinh). 2024 Dec 27;12(13):2412670. doi: 10.1002/advs.202412670 (PMC11967857; doi:10.1002/advs.202412670)
Supplement: Supplementary file 1 — Supporting Information [file ADVS-12-2412670-s001.docx]

**Metabolic Engineering of *Corynebacterium glutamicum* for High-Level Production of 1,5-Pentanediol, a C5 Diol Platform Chemical**

Sohn and Hwang *et al*.

**
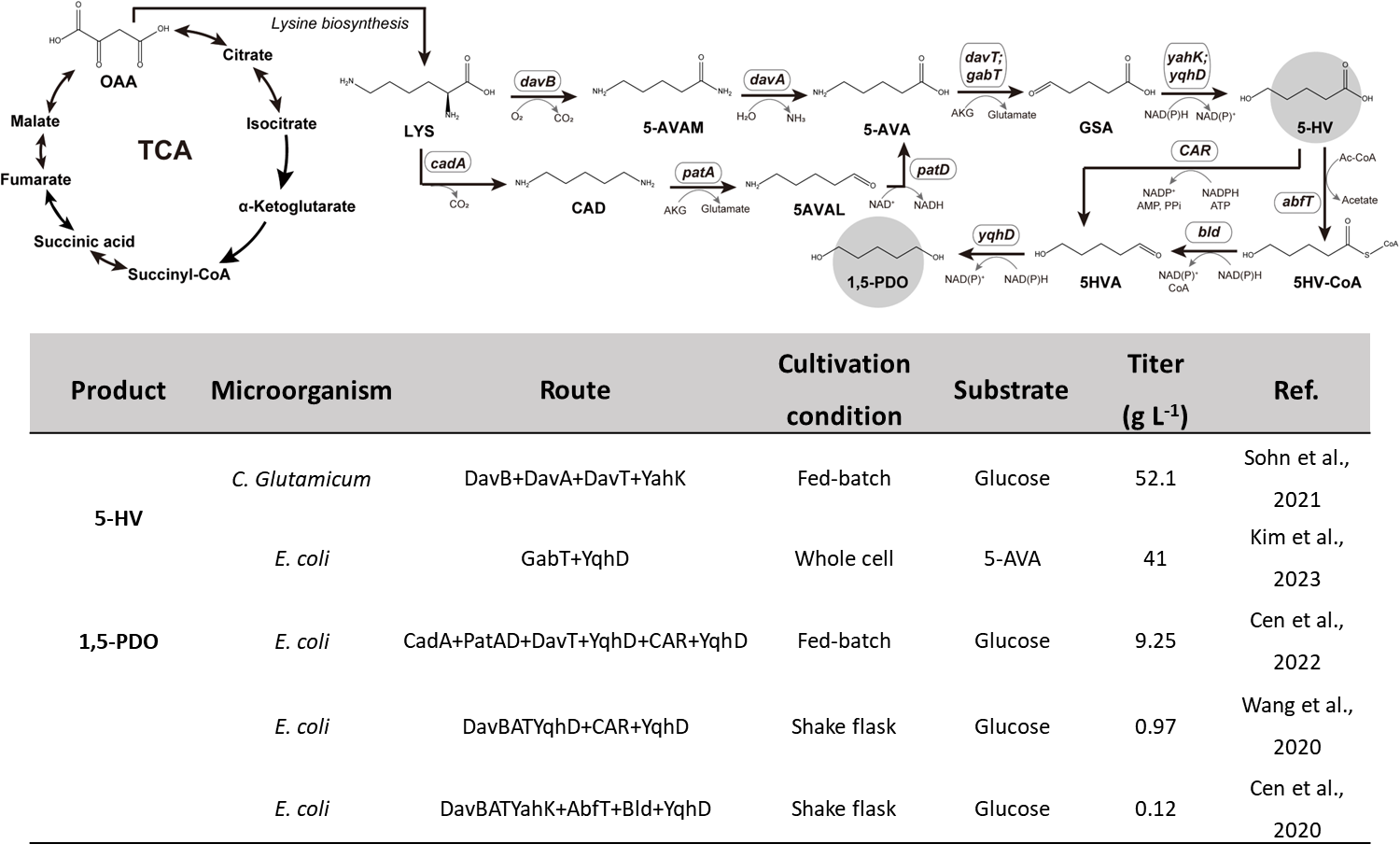
**

**Figure S1. Summary of previous studies for the microbial production of 5-HV and 1,5-PDO ^[1-5]^.** The abbreviations shown are as follows: GLU, glucose; PYR, pyruvate; OAA, oxaloacetate; LYS, l-lysine; 5-AVAM, 5-aminovaleramide; 5-AVA, 5-aminovaleric acid; GSA, glutarate semialdehyde; GTA, glutaric acid; 5-HV, 5-hydroxyvaleric acid; 5HV-CoA, 5-hydroxyvaleryl-CoA; 5HVA, 5-hydroxyvaleraldehyde; 1,5-PDO, 1,5-pentanediol; CAD, cadaverine; 5AVAL, 5-aminovaleraldehyde; *davB*, lysine monooxygenase; *davA*, 5-aminovaleramide amidohydrolase; *davT*, 5AVA aminotransferase; *yahK*, aldehyde reductase; *abfT*, CoA transferase; *bld*, aldehyde dehydrogenase; *yqhD*, aldehyde reductase; *cadA*, cis-aconitate decarboxylase; *patA*, putrescine transaminase; *patD*, ɤ-aminobutyraldehyde dehydrogenase.

**
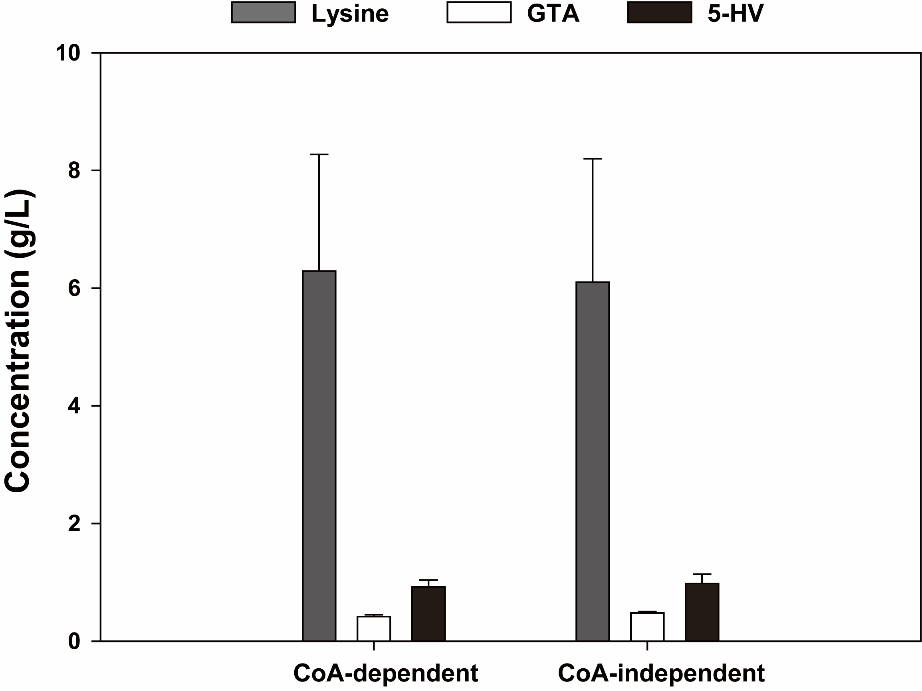
**

**Figure S2.** Flask cultivation of *C. glutamicum ΔgabD* (pCES208H30DavTYahKDavBhisA + pBL712H30AbfTBld^L273T^YqhD; CoA-dependent) and *C. glutamicum ΔgabD* (pCES208H30DavTYahKDavBhisA + pBL712H30MMAR2117PPtaseYqhD; CoA-independent) strains.

**
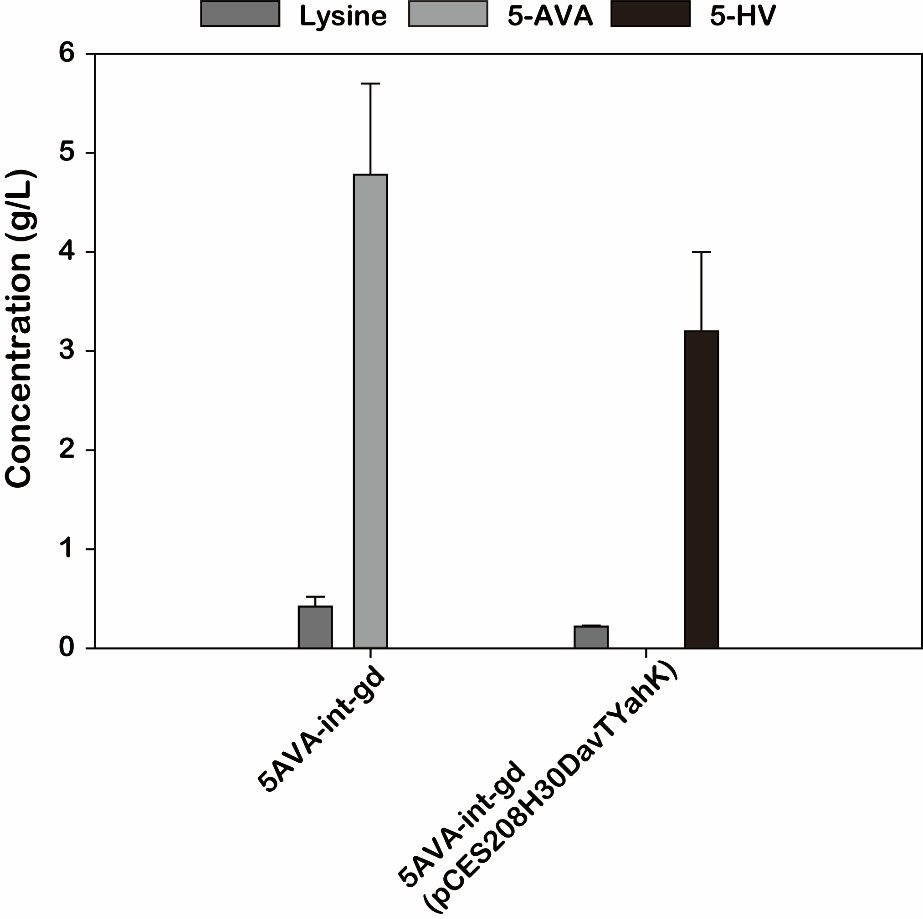
**

**Figure S3.** Flask cultivation of *C. glutaicum* 5AVA-int-gd and *C. glutaicum* 5AVA-int-gd (pCES208H30DavTYahK) strains.


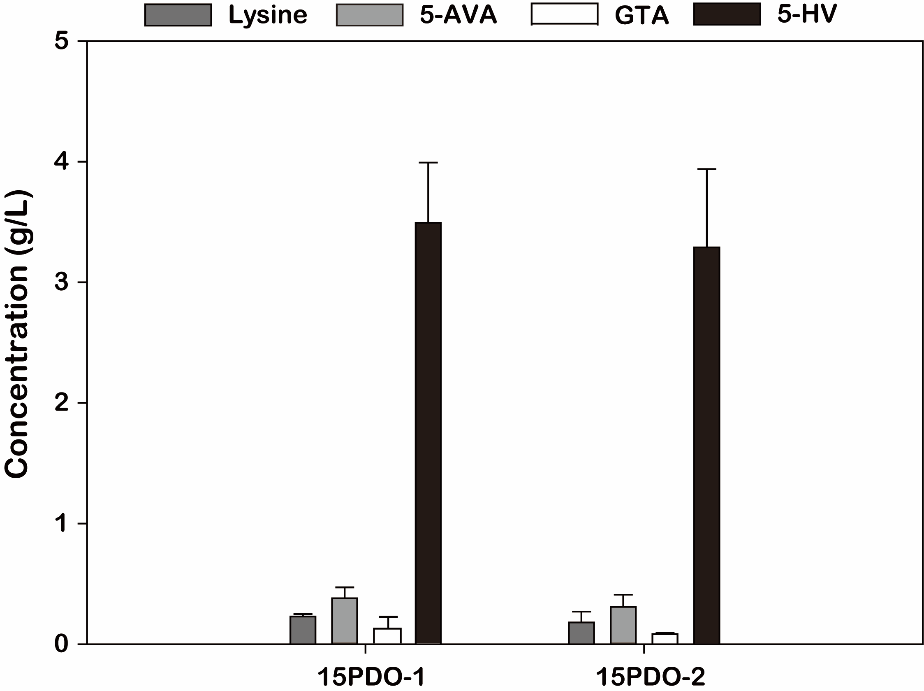


**Figure S4.** Flask cultivation of *C. glutamicum* 15PDO-1 and 15PDO-2 strains.


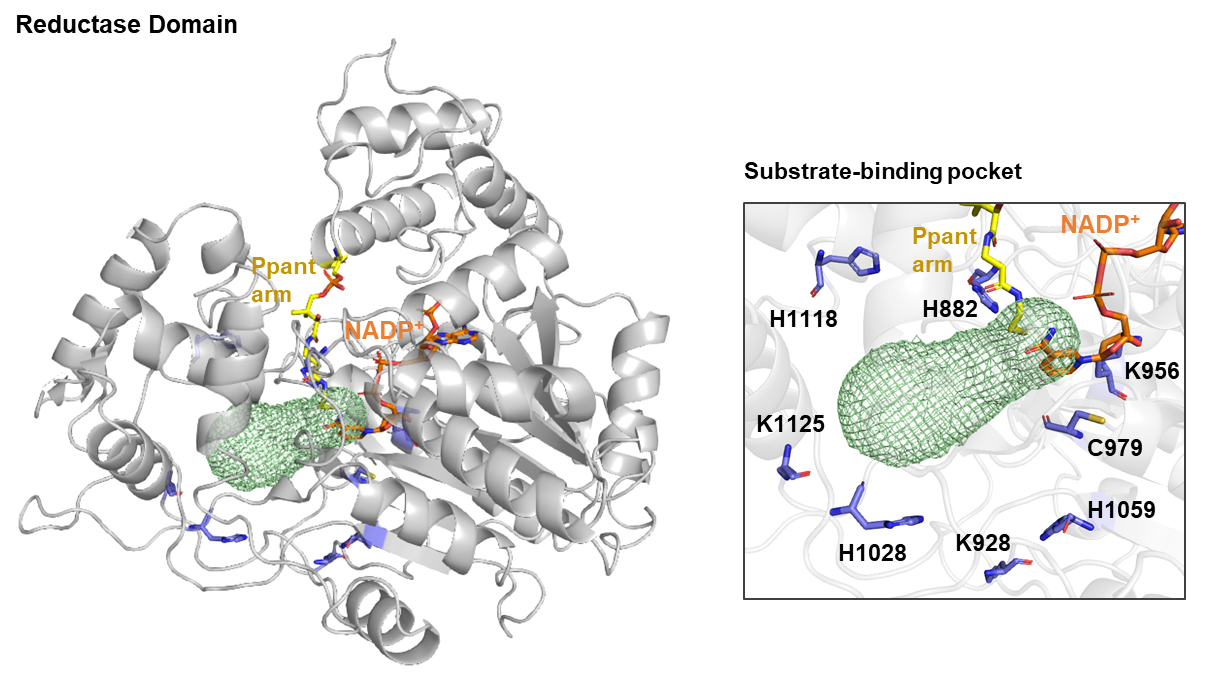


**Figure S5.** Structural analysis of PCP-R didomain of carboxylic acid reductase (MAP1040) modeled by AlphaFold2. Close-up view of the active site and substrate-binding cavity. The NADP cofactor is colored in orange and Ppant arm is in yellow.


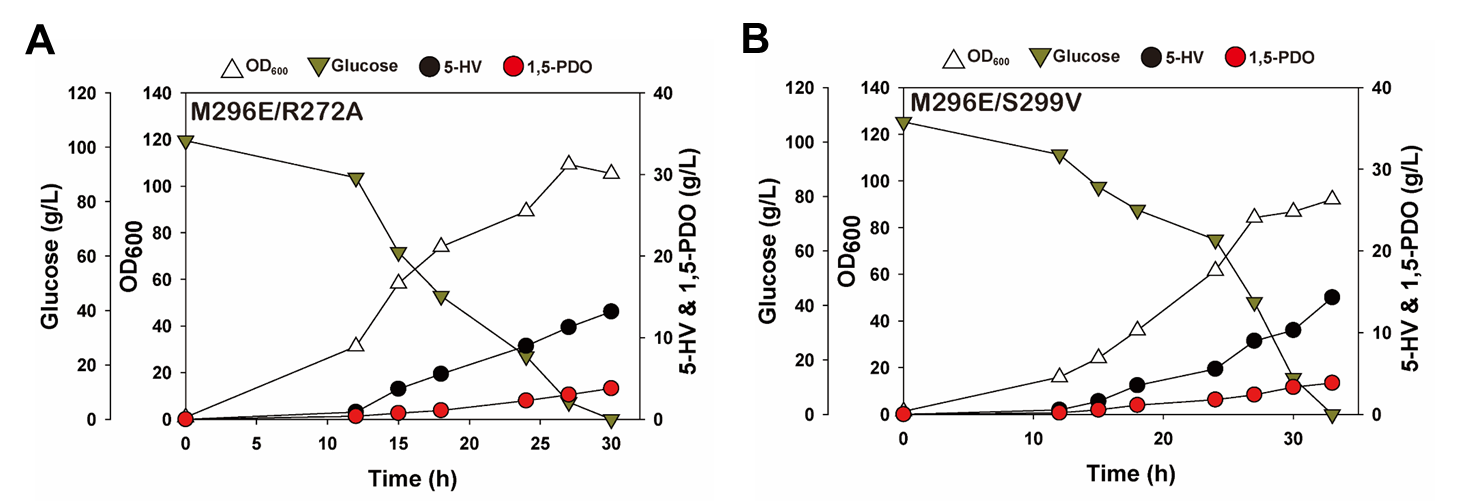


**Figure S6.** Batch fermentation of 15PDO-13(M296E/R272A) strain **(A)**, and 15PDO-13(M296E/S299V) strain **(B)**.


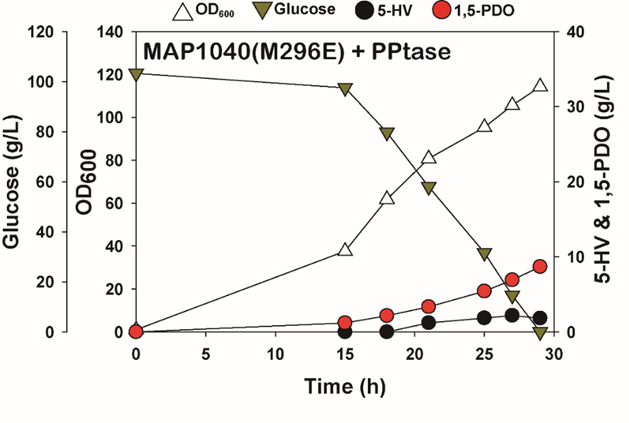


**Figure S7.** Batch fermentation of *C. glutamicum* 15PDO-13(M296E)-X strain.

**
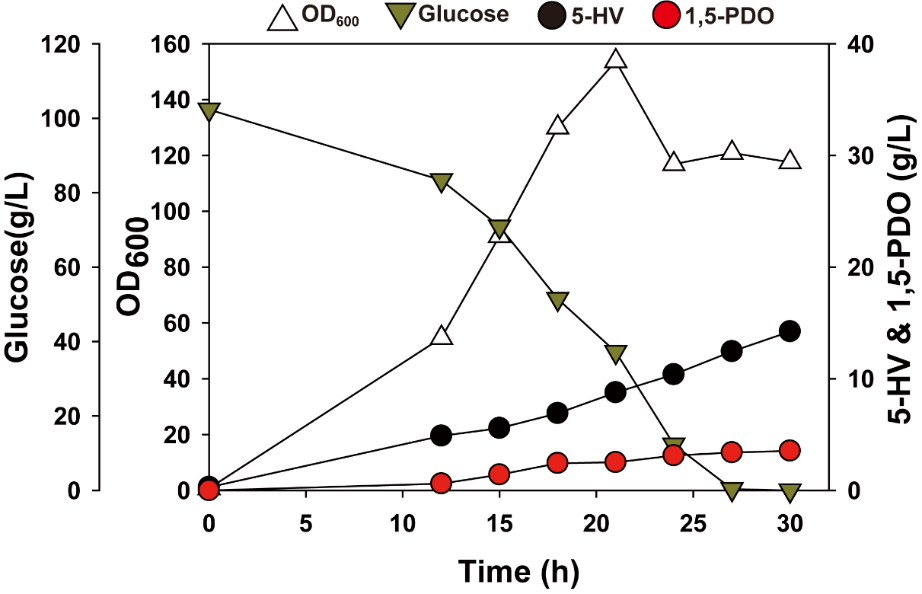
**

**Figure S8.** Batch fermentation of *C. glutamicum* 15PDO-13(M296E)-H strain for 1,5-PDO production.


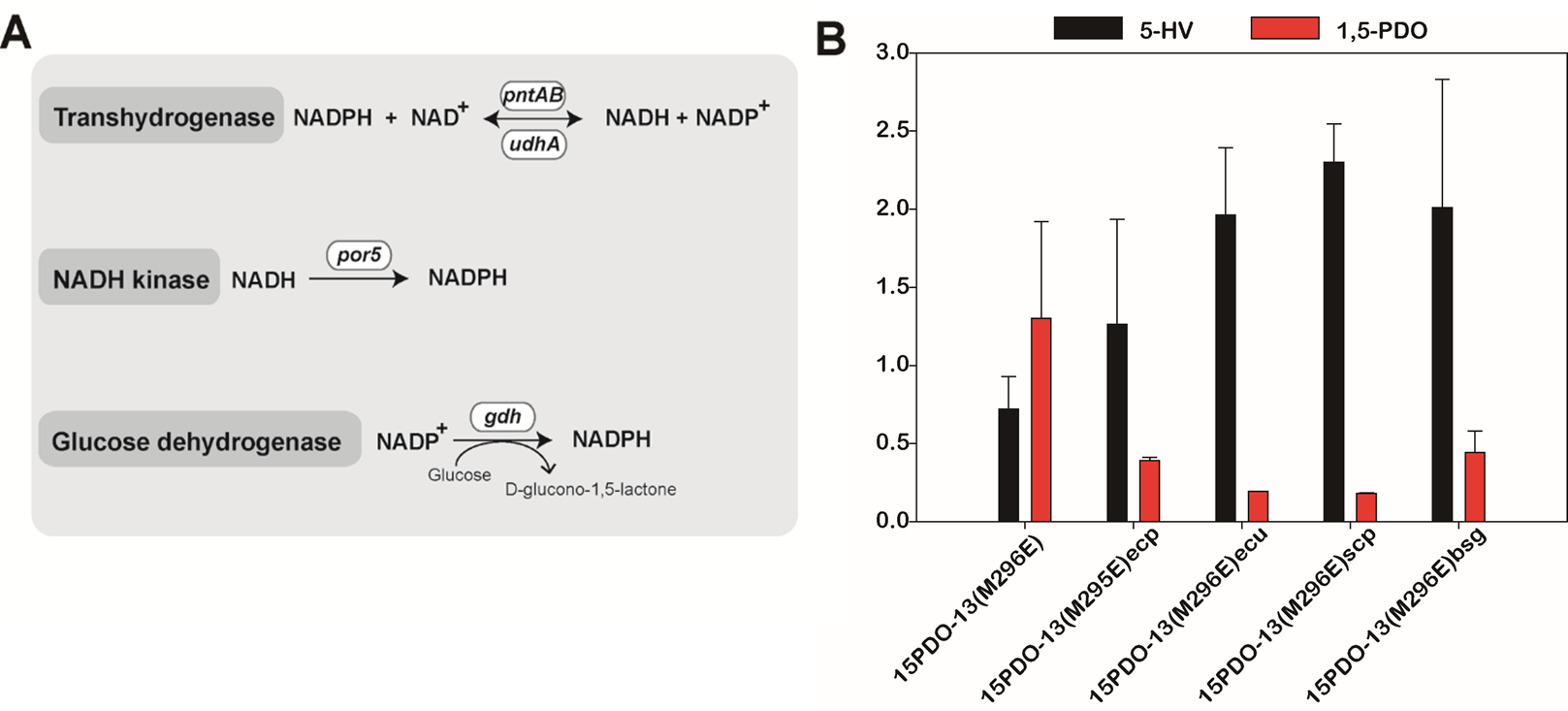


**Figure S9. (A)** Metabolic engineering strategies devised for the improvement of NADPH availability. **(B)** Flask cultivation of *C. glutamicum* 15PDO strains for 1,5-PDO production. All flask cultures were done in triplicates. The measurements were presented as mean ± standard deviation.

**Table S1.** CARs used in this study

| Strains | Protein name | Uniprot No. | Microorganism |
| --- | --- | --- | --- |
| 15PDO-1, 2, 3 | MMAR2117 | B2HN69 | *Mycobacterium marinum* |
| 15PDO-4 | MSM2108 | I7FIG7 | *Mycobacterium smegmatis* |
| 15PDO-5 | MAB2962 | B1MCR9 | *Mycobacterium abscessus* |
| 15PDO-6 | MAB4714 | B1MLD7 | *Mycobacterium abscessus* |
| 15PDO-7 | MSM5739 | A0R484 | *Mycobacterium smegmatis* |
| 15PDO-8 | MAB3367 | B1MDX4 | *Mycobacterium smegmatis* |
| 15PDO-9 | MSM2956 | A0QWI7 | *Mycobacterium smegmatis* |
| 15PDO-10 | MAB2963 | B1MCS0 | *Mycobacterium smegmatis* |
| 15PDO-11 | MSM5586 | I7GER2 | *Mycobacterium smegmatis* |
| 15PDO-12 | MMAR2936 | B2HE95 | *Mycrobacterium marinum* |
| 15PDO-13 | MAP1040 | Q741P9 | *Mycobacterium paratuberculosis* K-10 |
| 15PDO-14 | NBRGN_110_01750 | A0A034UK40 | *Nocardia brasiliensis* ATCC19296 |
| 15PDO-15 | NBGRN_060_00960 | A0A034UFC8 | *Nocardia brasiliensis* ATCC19296 |
| 15PDO-16 | NPHL210000_05340 | A0A5N5VEC4 | *Mycobacterium phlei* |

**Table S2.** Aldehyde reductases analyzed in this study

| Gene | Description | NAD | NADP | Label |
| --- | --- | --- | --- | --- |
| *ybbO* | NADP(+)-dependent aldehyde reductase | 0.0012 | 0.9987 | NADP^+^ |
| *ydfG* | 3-hydroxyacid dehydrogenase | 0.0015 | 0.9985 | NADP^+^ |
| *yghZ* | Aldo-keto reductase | 0.004 | 0.996 | NADP^+^ |
| *yafB* | Putative aldose reductase | 0.0014 | 0.9986 | NADP^+^ |
| *yjgB* | Predicted alcohol dehydrogenase | 0.0116 | 0.9884 | NADP^+^ |
| *yahK* | Aldehyde reductase | 0.0137 | 0.9864 | NADP^+^ |
| *yihU* | Sulfolactaldehyde | 0.9968 | 0.0031 | NAD^+^ |
| PP3340 | Aldo-keto reductase | 0.0133 | 0.987 | NADP^+^ |
| PS2058 | 3-oxoacyl-ACP reductase family protein | 0.0013 | 0.9986 | NADP^+^ |
| PA1146 | Iron-containing alcohol dehydrogenase | 0.9934 | 0.0066 | NAD^+^ |
| RHA07897 | Diacetyl reductase 3-reductase | 0.9986 | 0.0013 | NAD^+^ |
| *cpnD* | 5-hydroxyvalerate dehydrogenase | 0.9975 | 0.0025 | NAD^+^ |
| *gbd* | NAD-dependent 4-hydroxybutyrate dehydrogenase | 0.9974 | 0.0026 | NAD^+^ |
| *butA* | L-2,3-butanediol dehydrogenase/acetoin reductase | 0.9986 | 0.0014 | NAD^+^ |
| GOX1147 | Gamma-glutamyl phosphate reductase | 0.000764 | 0.999 | NADP^+^ |
| GOX1458 | Putative oxidoreductase | 0.0015 | 0.9985 | NADP^+^ |
| GOX0499 | Putative NAD-dependent aldehyde dehydrogenase | 0.125 | 0.8729 | NADP^+^ |
| GOX1122 | Putative NAD-dependent aldehyde dehydrogenase | 0.0861 | 0.9124 | NADP^+^ |
| GOX1801 | Putative oxidoreductase | 0.9657 | 0.0333 | NAD^+^ |
| GOX2181 | Putative polyol dehydrogenase | 0.9985 | 0.0015 | NAD^+^ |
| GOX1899 | Putative oxidoreductase | 0.0013 | 0.9987 | NADP^+^ |
| GOX2253 | Putative oxidoreductase | 0.000871 | 0.999 | NADP^+^ |
| GOX0878 | Putative oxidoreductase | 0.000884 | 0.999 | NADP^+^ |
| H16-A1168 | Dehydrogenase, related to short-chain alcohol dehydrogenase | 0.000997 | 0.999 | NADP^+^ |
| H16-B1297 | Short chain dehydrogenase | 0.0015 | 0.9985 | NADP^+^ |

**Table S3.** Strains and plasmids used in this study.

| **Name** | **Relevant genotype^a^** | **Reference** |
| --- | --- | --- |
| **Strains** |  |  |
| *E. coli* XL1-Blue | *recA1 endA1 gyrA96 thi-1 hsdR17 supE44 relA1 lac* [FA1*proAB lacI^q^ZΔM15* Tn*10* (Tet^R^)] | Stratagene^b^ |
| *C. glutamicum* PKC | An expired industrial l-lysine-producing strain | [6] |
| *C. glutamicum* Δ*gabD2* | *C. glutamicum* PKC Δ*gabD2* | [1] |
| *C. glutamicum* AVA-int-gd | *C. glutamicum* Δ*gabD2* with chromosomal deletion of *lysE* gene by integration of P_H30_DavB_His_A expression cassette | This study |
| *C. glutamicum* 15PDO-1 | *C. glutamicum* AVA-int-gd harboring pCES208H30DavTYahK and pBL712H30AbfTBld*YqhD | This study |
| *C. glutamicum* 15PDO-2 | *C. glutamicum* AVA-int-gd harboring pCES208H30DavTYahK and pBL712H30MMAR2117PPtaseYqhD | This study |
| *C. glutamicum* 5HV-int | *C. glutamicum* Δ*gabD2* with chromosomal deletion of *lysE* gene by integration of P_H30_DavB_His_A expression cassette and chromosomal deletion of *gabD3* gene by integration of P_H30_DavTYahK expression cassette | This study |
| *C. glutamicum* 15PDO-3 | *C. glutamicum* 5HV-int harboring pCES208H30MMAR2117PPtase and pBL712H30YqhD | This study |
| *C. glutamicum* 15PDO-4 | *C. glutamicum* 5HV-int harboring pCES208H30MSM2108PPtase and pBL712H30YqhD | This study |
| *C. glutamicum* 15PDO-5 | *C. glutamicum* 5HV-int harboring pCES208H30MAB2962PPtase and pBL712H30YqhD | This study |
| *C. glutamicum* 15PDO-6 | *C. glutamicum* 5HV-int harboring pCES208H30MAB4714PPtase and pBL712H30YqhD | This study |
| *C. glutamicum* 15PDO-7 | *C. glutamicum* 5HV-int harboring pCES208H30MSM5739PPtase and pBL712H30YqhD | This study |
| *C. glutamicum* 15PDO-8 | *C. glutamicum* 5HV-int harboring pCES208H30MAB3367PPtase and pBL712H30YqhD | This study |
| *C. glutamicum* 15PDO-9 | *C. glutamicum* 5HV-int harboring pCES208H30MSM2956PPtase and pBL712H30YqhD | This study |
| *C. glutamicum* 15PDO-10 | *C. glutamicum* 5HV-int harboring pCES208H30MAB2963PPtase and pBL712H30YqhD | This study |
| *C. glutamicum* 15PDO-11 | *C. glutamicum* 5HV-int harboring pCES208H30MSM5586PPtase and pBL712H30YqhD | This study |
| *C. glutamicum* 15PDO-12 | *C. glutamicum* 5HV-int harboring pCES208H30MMAR2936PPtase and pBL712H30YqhD | This study |
| *C. glutamicum* 15PDO-13 | *C. glutamicum* 5HV-int harboring pCES208H30MAP1040PPtase and pBL712H30YqhD | This study |
| *C. glutamicum* 15PDO-14 | *C. glutamicum* 5HV-int harboring pCES208H30NBRGN110PPtase and pBL712H30YqhD | This study |
| *C. glutamicum* 15PDO-15 | *C. glutamicum* 5HV-int harboring pCES208H30NBRGN060PPtase and pBL712H30YqhD | This study |
| *C. glutamicum* 15PDO-16 | *C. glutamicum* 5HV-int harboring pCES208H30MPHL21000PPtase and pBL712H30YqhD | This study |
| *C. glutamicum* 15PDO-13(M296E) | *C. glutamicum* 5HV-int harboring pCES208H30MAP1040(M296E)PPtase and pBL712H30YqhD | This study |
| *C. glutamicum* 15PDO-13(M422E) | *C. glutamicum* 5HV-int harboring pCES208H30MAP1040(M422E)PPtase and pBL712H30YqhD | This study |
| *C. glutamicum* 15PDO-13(R272A) | *C. glutamicum* 5HV-int harboring pCES208H30MAP1040(R272A)PPtase and pBL712H30YqhD | This study |
| *C. glutamicum* 15PDO-13(N465T) | *C. glutamicum* 5HV-int harboring pCES208H30MAP1040(N465T)PPtase and pBL712H30YqhD | This study |
| *C. glutamicum* 15PDO-13(M269F) | *C. glutamicum* 5HV-int harboring pCES208H30MAP1040(M269F)PPtase and pBL712H30YqhD | This study |
| *C. glutamicum* 15PDO-13(S299V) | *C. glutamicum* 5HV-int harboring pCES208H30MAP1040(S299V)PPtase and pBL712H30YqhD | This study |
| *C. glutamicum* 15PDO-13(M296E/R272A) | *C. glutamicum* 5HV-int harboring pCES208H30MAP1040(M296E/R272A)PPtase and pBL712H30YqhD | This study |
| *C. glutamicum* 15PDO-13(M296E/S299V) | *C. glutamicum* 5HV-int harboring pCES208H30MAP1040(M296E/S299V)PPtase and pBL712H30YqhD | This study |
| *C. glutamicum* 15PDO-13(M296E)-X | *C. glutamicum* 5HV-int harboring pCES208H30MAP1040(M296E/M412L)PPtase | This study |
| *C. glutamicum* 15PDO-13(M296E)-H | *C. glutamicum* 5HV-int harboring pHCPH30YqhD and pBL712H30CAR11(M296E)PPtaseYqhD | This study |
| *C. glutamicum* 15PDO-13(M296E)ecp | *C. glutamicum* 5HV-int harboring pCES208H30MAP1040(M296E)PPtase and pBL712H30YqhDPntAB | This study |
| *C. glutamicum* 15PDO-13(M296E)ecu | *C. glutamicum* 5HV-int harboring pCES208H30MAP1040(M296E)PPtase and pBL712H30YqhDUdhA | This study |
| *C. glutamicum* 15PDO-13(M296E)scp | *C. glutamicum* 5HV-int harboring pCES208H30MAP1040(M296E)PPtase and pBL712H30YqhDPos5 | This study |
| *C. glutamicum* 15PDO-13(M296E)bsg | *C. glutamicum* 5HV-int harboring pCES208H30MAP1040(M296E)PPtase and pBL712H30YqhDGDH | This study |
| *C. glutamicum* 15PDO-13(M296E)Y | *C. glutamicum* 5HV-int harboring pCES208H30MAP1040(M296E)PPtase and pBL712H30YihU | This study |
| *C. glutamicum* 15PDO-13(M296E)P | *C. glutamicum* 5HV-int harboring pCES208H30MAP1040(M296E)PPtase and pBL712H30PA1146 | This study |
| *C. glutamicum* 15PDO-13(M296E)R | *C. glutamicum* 5HV-int harboring pCES208H30MAP1040(M296E)PPtase and pBL712H30RHA07897 | This study |
| *C. glutamicum* 15PDO-13(M296E)C | *C. glutamicum* 5HV-int harboring pCES208H30MAP1040(M296E)PPtase and pBL712H30CpnD | This study |
| *C. glutamicum* 15PDO-13(M296E)Gb | *C. glutamicum* 5HV-int harboring pCES208H30MAP1040(M296E)PPtase and pBL712H30Gbd | This study |
| *C. glutamicum* 15PDO-13(M296E)B | *C. glutamicum* 5HV-int harboring pCES208H30MAP1040(M296E)PPtase and pBL712H30ButA | This study |
| *C. glutamicum* 15PDO-13(M296E)G18 | *C. glutamicum* 5HV-int harboring pCES208H30MAP1040(M296E)PPtase and pBL712H30GOX1801 | This study |
| *C. glutamicum* 15PDO-13(M296E)G21 | *C. glutamicum* 5HV-int harboring pCES208H30MAP1040(M296E)PPtase and pBL712H30GOX2181 | This study |
|  |  |  |
| *Plasmids* |  |  |
| pCES208H30GFP | pCES208 derivative; P_H30,_ eGFP; Km^R^ | [7] |
| pCES208H30DavB_His_A | pCES208 derivative; P_H30_, *P. putida* KT2440 *davBhisA;* Km^r^ | [8] |
| pCES208H30DavTYahK | pCES208 derivative; P_H30_, *P. putida* KT2440 *davT*, *E. coli yahK;* Km^r^ | This study |
| pBL712H30-MCS | pBL712 derivative; PH30; Sp^r^ | [1] |
| pBL712H30AbfTBld*YqhD | pBL712 derivative; P_H30_, *C. aminobutyricum abfT*, *C. saccharoperbutylacetonicum bld* (L273T), *E. coli yqhD*; Sp^r^ | This study |
| pBL712H30MMAR2117PPtaseYqhD | pBL712 derivative; P_H30_, *M. marinum* MMAR2117, *B. subtilis pptase, E. coli yqhD*; Sp^r^ | This study |
| pK19mobSacB-lysEFB | pK19mobSacB derivative; *lysE* deletion vector | [6] |
| pK19mobSacBLysE::H30DavB_His_A | pK19mobSacB derivative; P_H30_DavB_His_A integration vector; designed to integrate P_H30_DavB_His_A expression cassette into *lysE* site | This study |
| pK19mobSacBCg0067 | pK19mobSacB derivative; *cg0067* (*gabD3*) deletion vector | This study |
| pK19mobSacBCg0067::H30DavTYahK | pK19mobSacB derivative; P_H30_DavTYahK integration vector; designed to integrate P_H30_DavTYahK expression cassette into *cg0067* site | This study |
| pCES208H30-MCS | pCES208 derivative; PH_30_ Promoter, MCS, KmR | This study |
| pCES208H30MMAR2117PPtase | pCES208 derivative; P_H30_, *M. marinum* MMAR2117, *B. subtilis pptase, E. coli yqhD*; Km^r^ | This study |
| pCES208H30MSM2108PPTase | pCES208 derivative; P_H30_, *M. smegmatis* MSM2108, *B. subtilis pptase, E. coli yqhD*; Km^r^ | This study |
| pCES208H30MAB2962PPTase | pCES208 derivative; P_H30_, *M. abscessus* MAB2962, *B. subtilis pptase, E. coli yqhD*; Km^r^ | This study |
| pCES208H30MAB2714PPTase | pCES208 derivative; P_H30_, *M. abscessus* MAB2714, *B. subtilis pptase, E. coli yqhD*; Km^r^ | This study |
| pCES208H30MSM5739PPTase | pCES208 derivative; P_H30_, *M. smegmatis* MSM5739, *B. subtilis pptase, E. coli yqhD*; Km^r^ | This study |
| pCES208H30MAB3367PPTase | pCES208 derivative; P_H30_, *M. smegmatis* MAB3367, *B. subtilis pptase, E. coli yqhD*; Km^r^ | This study |
| pCES208H30MSM2956PPTase | pCES208 derivative; P_H30_, *M. smegmatis* MSM2956, *B. subtilis pptase, E. coli yqhD*; Km^r^ | This study |
| pCES208H30MSM2963PPTase | pCES208 derivative; P_H30_, *M. smegmatis* MSM2963, *B. subtilis pptase, E. coli yqhD*; Km^r^ | This study |
| pCES208H30MSM5586PPTase | pCES208 derivative; P_H30_, *M. smegmatis* MSM5586, *B. subtilis pptase, E. coli yqhD*; Km^r^ | This study |
| pCES208H30MMAR2936PPTase | pCES208 derivative; P_H30_, *M. marinum* MMAR2936, *B. subtilis pptase, E. coli yqhD*; Km^r^ | This study |
| pCES208H30MAP1040PPTase | pCES208 derivative; P_H30_, *Mycobacterium avium subsp.* paratuberculosi*s* K-10 MAP1040, *B. subtilis pptase, E. coli yqhD*; Km^r^ | This study |
| pCES208H30NBRGN110PPTase | pCES208 derivative; P_H30_, *N. brasiliensis* ATCC 19296 NBRGN_110_01750, *B. subtilis pptase, E. coli yqhD*; Km^r^ | This study |
| pCES208H30NBRGN060PPTase | pCES208 derivative; P_H30_, *N. brasiliensis* ATCC 19296 NBRGN_060_00960, *B. subtilis pptase, E. coli yqhD*; Km^r^ | This study |
| pCES208H30MPHL21000PPTase | pCES208 derivative; P_H30_, *M. phlei* MPHL21000_05340, *B. subtilis pptase, E. coli yqhD*; Km^r^ | This study |
| pBL712H30YqhD | pBL712 derivative; P_H30_, *E. coli yqhD*; Sp^r^ | This study |
| pCES208H30MAP1040(M296E)PPtase | pCES208 derivative; P_H30_, *M. avium* subsp. *paratuberculosis K-10 MAP1040* (M296E), *B. subtilis pptase, E. coli yqhD*; Km^r^ | This study |
| pCES208H30MAP1040(M422E)PPtase | pCES208 derivative; P_H30_, *M. avium* subsp. *paratuberculosis* K-10 MAP1040 (M424E), *B. subtilis pptase, E. coli yqhD*; Km^r^ | This study |
| pCES208H30MAP1040(R272A)PPtase | pCES208 derivative; P_H30_, *M. avium* subsp. *paratuberculosis* K-10 MAP1040 (R272A), *B. subtilis pptase, E. coli yqhD*; Km^r^ | This study |
| pCES208H30MAP1040(N465T)PPtase | pCES208 derivative; P_H30_, *M. avium* subsp. *paratuberculosis* K-10 MAP1040 (N465T), *B. subtilis pptase, E. coli yqhD*; Km^r^ | This study |
| pCES208H30MAP1040(M269F)PPtase | pCES208 derivative; P_H30_, *M. avium* subsp. *paratuberculosis* K-10 MAP1040 (M269F), *B. subtilis pptase, E. coli yqhD*; Km^r^ | This study |
| pCES208H30MAP1040(S299V)PPtase | pCES208 derivative; P_H30_, *M. avium* subsp. *paratuberculosis* K-10 MAP1040 (S299V), *B. subtilis pptase, E. coli yqhD*; Km^r^ | This study |
| pCES208H30MAP1040(M296E/R272A)PPtase | pCES208 derivative; P_H30_, *M. avium* subsp. *paratuberculosis* K-10 MAP1040 (M296E/R272A), *B. subtilis pptase, E. coli yqhD*; Km^r^ | This study |
| pCES208H30MAP1040(M296E/S299V)PPtase | pCES208 derivative; P_H30_, *M. avium* subsp. *paratuberculosis* K-10 MAP1040 (M296E/S299V), *B. subtilis pptase, E. coli yqhD*; Km^r^ | This study |
| pHCP | pCES-PLPV derivative; *parB* nonsense mutation, P_H36_, Km^r^ | [9] |
| pHCPH30-MCS | pHCP derivative; P_H30_; Km^r^ | This study |
| pHCPH30YqhD | pHCP derivative; P_H30_; *E. coli yqhD*; Km^r^ | This study |
| pBL712H30MAP1040(M296E)PPtaseYqhD | pBL712 derivative; P_H30_, *M. paratuberculosis* K-10 MAP1040 (M296E), *B. subtilis pptase, E. coli yqhD*; Sp^r^ | This study |
| pBL712H30YqhDPntAB | pBL712 derivative; P_H30_, *E. coli yqhD, pntAB*; Sp^r^ | This study |
| pBL712H30YqhDUdhA | pBL712 derivative; P_H30_, *E. coli yqhD, udhA*; Sp^r^ | This study |
| pBL712H30YqhDPos5 | pBL712 derivative; P_H30_, *E. coli yqhD, S. cerevisiae pos5*; Sp^r^ | This study |
| pBL712H30YqhDGDH | pBL712 derivative; P_H30_, *E. coli yqhD, B. subtilis* GDH; Sp^r^ | This study |
| pBL712H30YihU | pBL712 derivative; P_H30_, *E. coli yihU*; Sp^r^ | [1] |
| pBL712H30PA1146 | pBL712 derivative; P_H30_, *P. aeruginosa* PA1146; Sp^r^ | This study |
| pBL712H30RHA07897 | pBL712 derivative; P_H30_, *Rhodococcus* sp*.* RHA1 RHA07897; Sp^r^ | This study |
| pBL712H30CpnD | pBL712 derivative; P_H30_, *Clostridium aminovalericum*; Sp^r^ | [1] |
| pBL712H30Gbd | pBL712 derivative; P_H30_, *Ralstonia eutropha gbd*; Sp^r^ | [1] |
| pBL712H30ButA | pBL712 derivative; P_H30_, *C. glutamicum butA*; Sp^r^ | [1] |
| pBL712H30GOX1801 | pBL712 derivative; P_H30_, *Gluconobacter oxydans gox1801*; Sp^r^ | This study |
| pBL712H30GOX2181 | pBL712 derivative; P_H30_, *G. oxydans gox2181*; Sp^r^ | This study |

^a^Abbreviations: Tet, tetracycline; Km, kanamycin; Sp, spectinomycin; R, resistance.

^b^Strategene, Corp., La Jolla, CA

**Table S4.** Primers used in this study.

| **Primer** | **Sequence (5’-3’)** |
| --- | --- |
| DavT-F | GGATCCATGAGCAAAACCAACGAATCCTTG |
| DavT-R | AGATCTTTAGGCGATTTCAGCGAAGCAC |
| YahK-F | GGATCCAGGAGATATACATATGAAGATCAAAGCTGTTGGTGC |
| YahK-R | GCGGCCGCTTAAGTCTGTTAGTGTGCGATTATC |
| AbfT-F | GAATTCATGGATTGGAAGAAGATC |
| AbfT-R | GGTACCTTAGAATGCCGCGTTGAATC |
| Bld*-F | GGTACCAGGAGATATACATATGATCAAGGACACCCTG |
| Bld*-R | GGATCCTTAACCGGCTAACACGCAGCG |
| YqhD-pBL-F | GGATCCAGGAGATATACATATGAACAACTTTAATCTGCAC |
| YqhD-pBL-R | CCTGCAGGAGGAGATATACAT |
| MMAR2117-G-F | GCAGGAGTATATTGGAATTCATGTCGCCAATCACGCGTG |
| MMAR2117-G-R | CTTCATATGTATATCTCCT TTAGAGCAGGCCGAGTAG |
| PPtase-G-F | TAAAGGAGATATACATATGAAGATTTACGGAATT |
| PPtase-G-R | TTCATATGTATATCTCCTTTATAAAAGCTCTTCGTAC |
| YqhD-G-F | TATAAAGGAGATATACATATGAACAACTTTAATCTGC |
| YqhD-G-R | TAGAGGATCCCCGGGTACCTTAGCGGGCGGCTTCGTATATAC |
| H30operon-F | CTGCAGACTAAAGGGAACAAAAGCTG |
| H30operon-R | CTGCAGGGTCCACCTACAACAAAGCT |
| MMAR2117-F | GGATCCATGTCGCCAATCACGCGTG |
| MMR2117-R | ACTAGTTTAGAGCAGGCCGAGTAG |
| MSM2108-F | GGATCCATGTGGGACATGCTCTTC |
| MSM2108-R | ACTAGTTTACAACAGGCCGACCTTC |
| MAB2962-F | ACTAGTATGACCGTGACCAACGAAA |
| MAB2962-R | CCTGCAGGTTATAGGAGTCCGAGCTG |
| MAB4714-F | GGATCCATGACTGAAACGATCTCC |
| MAB4714-R | ACTAGTTTACACCAGGCCCAACAG |
| MSM5739-F | ACTAGTATGACCAGCGATGTTCAC |
| MSM5739-R | CCTGCAGGTTAGATCAGACCGAACTC |
| MAB3367-F | ACTAGTATGACGGCTGGTGCGGCG |
| MAB3367-R | CCTGCAGGTTACAGCAGCCTGTGTGC |
| MSM2956-F | ACTAGTATGACGATCGAAACGCGC |
| MSM2956-R | CCTGCAGGTTACAGCAATCCGAGCATC |
| MAB2963-F | GGATCCATGACGATCGACGCCACC |
| MAB2963-R | ACTAGTTAGAGTAACCCGAGCTG |
| MSM5586-F | ACTAGTATGCACCAGCTCACGGTC |
| MSM5586-R | CCTGCAGGTTAGATCAGACCGAACTC |
| MMAR2936-F | GGATCCATGTCAATTACCTGTGTG |
| MMAR2936-R | ACTAGTTTAAGCCAGGCCGAGAAG |
| MAP1040-F | ACTAGTATGTCGACTGCCACCCATG |
| MAP1040-R | CCTGCAGGTTAGAGCAGCCCGAGCAG |
| NBRGN110-F | ACTAGTATGGAGCGCAAGGCGGAAG |
| NBRGN110-R | CCTGCAGGTTACAGCAGGTTTCGCAAT |
| NBRGN060-F | GGATCCATGACAGATGTAGAGGTAG |
| NBRGN060-R | ACTAGTTTACAGTCCGAGGTGCTCCAG |
| MPHL-F | ACTAGTATGGCATCAGAATCCCGTG |
| MPHL-R | CCTGCAGGTTACAGCCCGAGCAGCCGCAGATCG |
| PPtase-F | CCTGCAGGTCTAGATAACTTTAAGAAGGAGATATACATGAAGATTTACGGAATTT |
| PPtase-R | CATATGTTATAAAAGCTCTTCGTAC |
| MAP1040-M296E-F | CACGTGGAAGGCCGAAGCATCCTCTAC |
| MAP1040-M296E-R | TCGGCCTTCCACGTGGCTCATCGGCATG |
| MAP1040-M422E-F | GCCGGAGAAGTGTTGTTCGACGGGGAG |
| MAP1040-M422E-R | CAACACTTCTCCGGCCTCGGTGGAGC |
| MAP1040-R272A-F | GATGTGGCGCGCAGGCAGCAAGAAC |
| MAP1040-R272A-R | TTGCCGACGTTGCTCTGTG |
| MAP1040-N465T-F | ACCGAGACCATGTTCCCGGGCTACTACAAG |
| MAP1040-N465T-R | CATGGTCTCGGTGCGCAGCAGCAGCTCGCCGCGCGGATGCG |
| MAP1040-M269F-F | CGTCGGCAAGTTCTGGCGCCGCG |
| MAP1040-M269F-R | TTGCTCTGTGGGTACATCGC |
| MAP1040-S299V-F | GGCCGAGTTATCCTCTACGGCACGCTGGG |
| MAP1040-S299V-R | GAGGATAACTCGGCCCATCACGTGGCTC |
| MAP1040-M296E/S299V-F | CACGTGGAAGGCCGAGTTATCCTCTACGGCACGCTGGG |
| MAP1040-M296E/S299V-R | GAGGATAACTCGGCCTTCCACGTGGCTCATCGGCATG |
| pHCP-H30-MCS-F | GGTACCAAAGTAACTTTTCGGTTAAGG |
| YqhD-pHCP-F | GGATCCATGAACAACTTTAATCTGCACACCC |
| YqhD-pHCP-R | GCGGCCGCTTAGCGGGCGGCTTCGTATATAC |
| MAP1040-G-F | TTGGTTGGGCAGGAGTATATTGGAATTCATGTCGACTGCCACCCATGACGAACGACTC |
| MAP1040-G-R | ACATCACCGACCTGCAGCTGCTCGGGCTGCTCTAAAGGAGATATACATATGAAGATTTACGGAATTTATATGGAC |
| MAP1040-PPtase-G-F | GTCCATATAAATTCCGTAAATCTTCATATGTATATCTCCTTTAGAGCAGCCCGAGCAGCTGCAGGTCGGTGATGT |
| PntA-F | GGTACCAGGAGATATACATATGCGAATTGGCATACCAAG |
| PntA-R | GGATCCTTAATTTTTGCGGAACATTTTC |
| PntB-F | GGATCCAGGAGATATACATATGTCTGGAGGATTAGTTAC |
| PntB-R | CCTGCAGGTTACAGAGCTTTCAGGATTGCATCCAC |
| UdhA-F | GGTACCAGGAGATATACATATGCCACATTCCTACGATTAC |
| UdhA-R | GGATCCTTAAAACAGGCGGTTTAAAC |
| Pos5-F | GGTACCAGGAGATATACATATGTTTGTCAGGGTTAAATTG |
| Pos5-R | GGATCCTTAATCATTATCAGTCTGTCTC |
| GDH-F | GGTACCTTAGGCGCTGAAGCCGCCGTCGATGGTCAG |
| GDH-R | GGATCCTTAACCGCGGCCTGCCTGGAATG |
| PA1146-F | GAATTCATGAGCGACCTGCATTACTG |
| PA1146-R | GGTACCTTAGGCGAGAGTCCCGGCCACCG |
| RHA07897-F | GAATTCATGAGCGACCTGCATTACTG |
| RHA07897-R | GGTACCTTAGGCGAGAGTCCCGGCCACCG |
| GOX1801-F | TCTAGAAGGAGATATACATATGTCGAGTCCAAAGATCG |
| GOX1801-R | CCTGCAGGTTATTTATGGGGAAGATTGG |
| GOX2181-F | GAATTCATGTACATGGAAAAACTCCG |
| GOX2181-R | GGTACCTTACCAGACGGTGAAGCC |
| Cg00671-F | AGTGACCATCGGCGGAGTGTTCG |
| Cg00671-R | GGGGCAGATGTCTAGAGATGCGTTATTTTCCTTCAC |
| Cg00672-F | ATAACGCATCTCTAGACATCTGCCCCTTTACAAATCC |
| Cg00672-R | CCTGCCATCCAGTCGGCATAC |
| Cg0067-F | AAGCTTAGTGACCATCGGCGGAGTGTTCG |
| Cg0067-R | GAATTCCCTGCCATCCAGTCGGCATAC |

**Supplementary Method 1. Construction of plasmids used in this study**

PCR was carried out using a C1000 Thermal Cycler (Bio-Rad, Hercules, CA, USA), with Pfu polymerase obtained from Elpis. Restriction endonucleases were sourced from New England Biolabs (NEB).

To construct pCES208H30DavTYahK, the *yahK* gene was PCR-amplified from *E. coli* genomic DNA using YahK-F/R primers, each containing a 5' ribosomal binding site extension. All subsequent primer sets used in this study also included this 5' extension. The amplified fragments were gel-purified, digested with *Bam*HI and *Not*I, and ligated into *Bam*HI/*Not*I-digested pCES208H30GFP ^[7]^, yielding pCES208H30YahK. Next, the *davT* gene was PCR-amplified from *Pseudomonas putida* KT2440 genomic DNA using DavT-F/R primers. The fragment was digested with *Bam*HI and *Bgl*II and ligated into *Bam*HI-digested pCES208H30YahK, generating pCES208H30DavTYahK.

For the construction of pBL712H30AbfTBld*YqhD, synthetic *abfT* (from *Clostridium aminobutyricum*) and *bld*(L273T) (from *Clostridium saccharoperbutylacetonicum*) genes were obtained from Cure Bio (Seoul, Korea). These genes were amplified using AbfT-F/R and Bld*-F/R primers, respectively. The *abfT* gene was ligated into *Eco*RI/*Kpn*I-digested pBL712H30-MCS^1^, yielding pBL712H30AbfT. The *bld*(L273T) gene was then cloned into *Kpn*I/*Bam*HI-digested pBL712H30AbfT, generating pBL712H30AbfTBld*. Finally, the *yqhD* gene, amplified from *E. coli* genomic DNA using YqhD-pBL-F/R primers, was cloned into *Bam*HI/*Sbf*I-digested pBL712H30AbfTBld*, forming pBL712H30AbfTBld*YqhD.

All CAR and PPtase genes ^[10-11]^ used in this study were kindly provided by Prof. Alexander F. Yakunin. For the construction of pBL712H30MMAR2117PPtaseYqhD, PCR amplification of *Mycobacterium marinum* MMAR2117 and *Bacillus subtilis* PPtase was carried out using MMAR2117-G-F/R and PPtase-G-F/R primers, respectively. The *yqhD* gene was also amplified using YqhD-G-F/R primers. All three fragments were assembled into *Eco*RI/*Kpn*I-digested pBL712H30-MCS using Gibson assembly.

To construct pCES208H30-MCS, a multiple cloning site (MCS) sequence (*ggatccg*agctc*actagt*cgg*caattg*tctaga*cctgcagg*cccgtc*catatg*gaccat*gcggccgc*) was inserted into *Bam*HI/*Not*I-digested pCES208H30GFP. The *B. subtilis* PPtase gene was amplified using PPtase-F/R primers and cloned into *Sbf*I/*Nde*I-digested pCES208H30-MCS, yielding pCES208H30PPtase. Various CAR genes (MMAR2117, MSM2108, MAB4714, MAB2963, MMAR2936, NBRGN060, MPHL) were amplified by PCR with specific primers and cloned into *Bam*HI/*Spe*I-digested pCES208H30PPtase, generating the corresponding pCES208H30-CAR-PPtase plasmids. Similarly, genes (MAB2962, MSM5739, MAB3367, MSM2956, MSM5586, MAP1040, NBRGN110) were cloned into *Spe*I/*Sbf*I-digested pCES208H30PPtase, producing additional pCES208H30-CAR-PPtase constructs.

To introduce point mutations into the MAP1040 gene, the upstream and downstream regions flanking the target base pairs were PCR-amplified using primer pairs MAP1040-F/MAP1040-Mut (e.g., M296E)-R and MAP1040-Mut (e.g., M296E)-F/MAP1040-R. The upstream and downstream fragments were combined via overlap extension PCR, yielding single linear fragments with mutations such as M296E, M422E, R272A, N465T, M269F, S299V, M296E/R272A, and M296E/S299V. The resulting fragments were digested with *Spe*I/*Sbf*I and ligated into *Spe*I/*Sbf*I-digested pCES208H30PPtase, constructing pCES208H30MAP1040(Mut)PPtase.

To construct pHCPH30-MCS, the PH30-MCS region was amplified from pCES208H30-MCS using pHCP-H30-MCS-F and H30operon-R primers, digested with *Kpn*I/*Not*I, and ligated into *Kpn*I/*Not*I-digested pHCP plasmid ^[9]^. The *yqhD* gene was then amplified using YqhD-pHCP-F/R primers and cloned into *Bam*HI/*Sbf*I-digested pHCPH30-MCS, generating pHCPH30YqhD.

To generate pBL712H30MAP1040(M296E)PPtaseYqhD, the mutated MAP1040(M296E) and PPtase genes were amplified from pCES208H30MAP1040(M296E)PPtase using MAP1040-G-F/R and MAP1040-PPtase-G-F/PPtase-G-R primers. The *yqhD* gene was amplified from *E. coli* and all fragments were assembled into *Eco*RI/*Kpn*I-digested pBL712H30-MCS by Gibson assembly.

For pBL712H30YqhDPntAB, *pntA* and *pntB* genes were amplified from *E. coli* genomic DNA using PntA-F/R and PntB-F/R primers, respectively, and sequentially inserted into *Kpn*I/*Bam*HI and BamHI/SbfI-digested pBL712H30YqhD. Similarly, *udhA*, *pos5*, and *gdh* genes were amplified from *E. coli*, *Saccharomyces cerevisiae*, and *Bacillus subtilis*, respectively, and cloned into *Kpn*I/*Bam*HI-digested pBL712H30YqhD, generating pBL712H30YqhDUdhA, pBL712H30YqhDPos5, and pBL712H30YqhDGDH.

For screening aldehyde reductases, PA1146 from *Pseudomonas aeruginosa* and RHA07897 from *Rhodococcus* sp. RHA1, provided by Prof. Yakunin ^[10]^, were amplified using PA1146-F/R and RHA07897-F/R primers and cloned into *Eco*RI/*Kpn*I-digested pBL712H30-MCS, generating pBL712H30PA1146 and pBL712H30RHA07897. Additionally, GOX1801 and GOX2181 from *Gluconobacter oxydans* 621H were amplified using GOX1801-F/R and GOX2181-F/R and cloned into *Xba*I/*Sbf*I and *Eco*RI/*Kpn*I-digested pBL712H30-MCS, respectively, creating pBL712H30GOX1801 and pBL712H30GOX2181.

**Supplementary Method 2. Construction of engineered *C. glutamicum* strains**

Chromosomal gene deletions and integration of gene expression cassettes were performed via homologous recombination using plasmid pK19mobSacB. The plasmid pK19mobSacBLysE::H30DavBHisA was designed to integrate an expression cassette, comprising a synthetic promoter, *P. putida* *davB_His_A* genes, and a terminator (*PH30DavBHisA*), into the *lysE* gene. The expression cassette (*P_H30_DavBHisA*) was amplified from pCES208H30DavBHisA ^[8]^ using the H30operon-F and H30operon-R primers and inserted into the pK19mobSacB-lysEFB plasmid ^[6]^ at the *Pst*I site.

To construct pK19mobSacBCg0067::H30DavTYahK, an expression cassette containing a synthetic promoter, *P. putida* *davT*, *E. coli* *yahK* genes, and a terminator (*P_H30_DavTYahK*), was integrated into the *cg0067* gene. Initially, a 500-bp upstream region of the target gene was PCR-amplified from the chromosomal DNA of *C. glutamicum* PKC using Cg00671-F and Cg00671-R primers. A 500-bp downstream region was similarly amplified using Cg00672-F and Cg00672-R primers. The two fragments were then combined by overlap extension PCR, yielding a 1000-bp fragment flanking the *cg0067* gene. This fragment was cloned into pK19mobSacB between *Hin*dIII and *Eco*RI sites, forming pK19mobSacBCg0067. The expression cassette (*P_H30_DavTYahK*) was amplified from pCES208H30DavTYahK using H30operon-F and H30operon-R primers and inserted into pK19mobSacBCg0067 at the *Xba*I site.

The integration of expression cassettes in *C. glutamicum* was performed using a *sacB*-based genetic engineering method. Cells were transformed via electroporation with either pK19mobSacBLysE::H30DavBHisA or pK19mobSacBCg0067::H30DavTYahK, and plated on RG agar plates containing kanamycin (Km). After 48 hours of incubation at 30°C, five single colonies were selected and inoculated into 15 mL test tubes containing 2 mL RG medium. Cultures were incubated in a shaking incubator at 250 rpm for 12 hours at 30°C. The cells were then streaked onto LB agar plates containing 15% (w/v) sucrose and incubated for another 48 hours at 30°C. Colonies growing on sucrose-containing LB agar were screened for loss of Km resistance, and successful genetic modifications were confirmed via PCR or sequencing. Using this *sacB*-based genetic engineering method, strains AVA-int-gd and 5HV-int were constructed with the respective pK19mobSacB-series vectors.

**Supplementary references**

1. Sohn, Y. J. et al. Fermentative high-level production of 5-hydroxyvaleric acid by metabolically engineered *Corynebacterium glutamicum*. *ACS Sustain. Chem. Eng.* **9**, 2523-2533 (2021)
2. Kim, B. et al. Complementation of reducing power for 5-hydroxyvaleric acid and 1, 5-pentanediol production via glucose dehydrogenase in *Escherichia coli* whole-cell system. *Enzyme Microb. Technol.* **170**, 110305 (2023)
3. Cen, X. et al. Metabolic engineering of *Escherichia coli* for high production of 1, 5-pentanediol via a cadaverine-derived pathway. *Metab. Eng*. **74**, 168-177 (2022)
4. Wang, J. et al. Bacterial synthesis of C3-C5 diols via extending amino acid catabolism. *Proc. Natl. Acad. Sci.* **117**, 19159-19167 (2020)
5. Cen, X. et al. Metabolic engineering of *Escherichia coli* for de novo production of 1, 5-pentanediol from glucose*. ACS Synth. Biol.* **10**, 192-203 (2020)
6. Kim, H. T. et al. Metabolic engineering of *Corynebacterium glutamicum* for the high-level production of cadaverine that can be used for the synthesis of biopolyamide 510. *ACS Sustain. Chem. Eng*. **6**, 5296-5305 (2018)
7. Yim, S. S. et al. Isolation of fully synthetic promoters for high‐level gene expression in *Corynebacterium glutamicum*. *Biotechnol. Bioeng.* **110**, 2959-2969 (2013)
8. Sohn, Y. J. et al. Development of a bio-chemical route to C5 plasticizer synthesis using glutaric acid produced by metabolically engineered *Corynebacterium glutamicum*. *Green Chem*. **24**, 1590-1602 (2022)
9. Choi, J. W., Yim, S. S. & Jeong, K. J. Development of a high-copy-number plasmid via adaptive laboratory evolution of *Corynebacterium glutamicum*. *Appl. Microbiol. Biotechnol*. **102**, 873-883 (2018).
10. Khusnutdinova, A. N. et al. Exploring bacterial carboxylate reductases for the reduction of bifunctional carboxylic acids. *Biotechnol. J.* **12**, 1600751 (2017)
11. Fedorchuk, T. P. et al. One-pot biocatalytic transformation of adipic acid to 6-aminocaproic acid and 1, 6-hexamethylenediamine using carboxylic acid reductases and transaminases. *J. Am. Chem. Soc.* 142, 1038-1048 (2019)
